# Development of a microwave-assisted sustainable conversion of furfural hydrazones to functionalised phthalimides in ionic liquids†

**DOI:** 10.1039/c8ra03895c

**Published:** 2018-06-20

**Authors:** Valerija Karaluka, Kengo Murata, Shinto Masuda, Yuto Shiramatsu, Takuji Kawamoto, Helen C. Hailes, Tom D. Sheppard, Akio Kamimura

**Affiliations:** Department of Applied Chemistry, Yamaguchi University Ube 755-8611 Japan ak10@yamaguchi-u.ac.jp; Department of Chemistry, University College London 20 Gordon Street London WC1H 0AJ UK

## Abstract

A sustainable synthetic procedure to convert furfural hydrazones into functionalised phthalimides was developed. The reaction was performed in a microwave using a hydrophilic ionic liquid, [bmim][Cl], as the solvent which could be readily recovered by a simple extraction. The ionic liquid was successfully recycled with no significant loss in product yields.

## Abstract 2

A sustainable synthetic procedure to convert furfural hydrazones into functionalised phthalimides in ionic liquids was developed.

## Graphical abstract

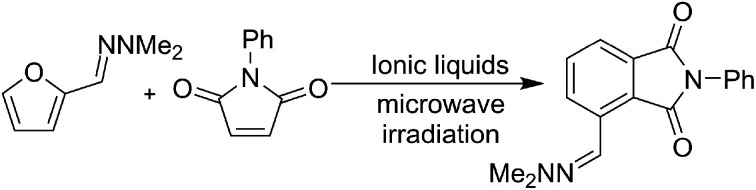

## Introduction

Increasing awareness of the negative impact that the use of non-renewable petrochemicals has on the environment has led the scientific community to investigate greener, more sustainable alternatives. For this reason, the use of biomass waste has been proposed as a potential feedstock for the generation of valuable chemicals and fuel.^1–5^ Furfural and HMF can be obtained from lignocellulosic biomass through the acid-catalysed dehydration of sugars.^2,3,6^ They have been reported as precursors to a number of valuable chemical products ranging from medicinally relevant compounds with furan scaffolds to cycloaddition products including poly-substituted benzenes.^4–9^ The use of furan and its derivatives in Diels–Alder cyclisations to access commodity aromatics (*e.g.* toluene, xylene, benzoic acid) has been widely investigated.^5,10,11^ Although the cycloaddition–aromatisation reaction of furans represents an atom economical strategy, as well as a green route to aromatic compounds because it only releases water as a waste by-product, many of the procedures reported to date employ relatively harsh conditions such as high temperatures/pressures, or require a large excess of the furan to achieve high conversions.^4,5,10^ Recently, procedures employing biomass-derived compounds in tandem with green solvents have been reported.^8,12–15^ Amongst them, we developed a one-pot protocol for the synthesis of polysubstituted aromatic compounds from furfural in water.^4^ This procedure is completely performed in water and avoids the use of any organic solvent in the extraction or purification steps. However, in some cases direct crystallisation of the product from the reaction mixture did not readily take place, and purification after extraction with organic solvents was necessary. In addition, where substrates are poorly soluble in water the reaction does not proceed efficiently. In this new study, we wished to explore the development of a more general protocol applicable to more hydrophobic compounds using ionic liquids as the medium for the reaction.

Ionic liquids are recognized as new green solvents for chemical transformations,^16–18^ because of their low volatility, compatibility with catalysts, ability to dissolve reactants, and their unique physical properties which often enable them to catalyse organic reactions.^16,19–21^ Additionally, it has been reported that reactions in ionic liquids were efficiently accelerated by microwave heating because very rapid heating of IL to high temperatures was easily achieved. This advantage is mainly because of dipolar rotation and ionic conduction effects derived from the use of ionic liquids.^22–30^ For example, we have recently demonstrated the successful utilisation of ionic liquids for; (i) depolymerisation of plastics,^31,32^ (ii) cellulolysis^33^ using the hydrophobic IL [PP13][NTf_2_], and (iii) conversion of sorbitol to isosorbide using [TMPA][NTf_2_].^34^

The ultimate goal of this current study is to develop a sustainable route for the conversion of biomass waste to useful aromatic compounds using ionic liquids. Herein, we report the use of an ionic liquid, [bmim][Cl], as an alternative green solvent for the synthesis of substituted phthalimides from furfurals. We also demonstrate the recyclability of [bmim][Cl] in the reaction.

## Results and discussion

We initially sought to identify suitable ionic liquids for the synthesis of phthalimides from furfural hydrazone 3a (Table 1). A variety of hydrophilic (entries 1, 2, 7, 8) and hydrophobic (entries 3–6) ionic liquids were examined. We first examined conventional heating of the reaction, but the reaction progressed slowly and the desired product 3a was obtained in only 32% yield (entry 1). To increase the reaction rate, microwave heating was employed. The IL [bmim][BF_4_] was found to be unsuitable for the reaction due to difficulties observed in the isolation of the product (entry 2), and the low recovery of the IL. We envisaged that [bmim][NTf_2_] might be effective at mediating the reaction due to its high ionicity.^35,36^ Although [bmim][NTf_2_] was found to give the best yield of 61% (entry 3), separation of the IL from the product was difficult because of its hydrophobicity, and this required copious washes with organic solvents, which was against the purpose of developing a sustainable environmentally friendly procedure. Similarly, [TMPA][NTf_2_] and [PP13][NTf_2_] resulted in good yields of 3a, but the recovery of the IL was not satisfactory (entries 4 and 5, respectively). The IL [DEME][NTf_2_] was a poor solvent for the reaction with only 32% yield of the product obtained (entry 6). We then examined the hydrophilic ionic liquid [bmim][Cl]. The reaction progressed well with microwave heating, and complete separation of the IL was accomplished by washing the organic layers three times with water, although the yield of 3a remained at 35% (entries 7). To improve the yield of 3a, we examined the use of freshly prepared hydrazone 1a. Compound 3a was then isolated in 83% yield without contamination of the IL (entry 8). Pleasingly, 99% of the IL was recovered after the work-up. The product 3a was isolated by recrystallisation from an acetone/water mixture without the need for chromatographic purification. It was considered that the lower yields initially observed during the formation of 3a could be attributed to contamination with *N*,*N*-dimethylaminohydrazine, liberated from hydrazone 1a on storage. Indeed, the cycloaddition reaction became sluggish when 10 mol% of dimethlhydrazine was added and the yield of compound 3a decreased to less than 10% (Table 1 footnote *d*). We think that contamination with small amounts of *N*,*N*-dimethylhydrazine in ionic liquids could progress some other reactions, possibly conjugate addition to 2a, preventing the formation of 3a in high yield.

**Table 1:** The Diels–Alder reaction of hydrazone 1a with maleimide 2

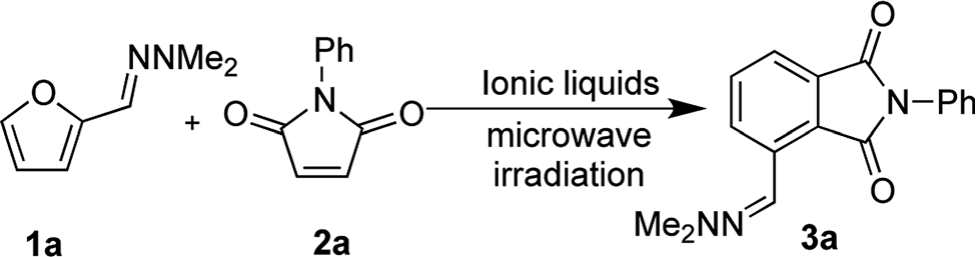					
Entry	Ionic liquids	Temp (°C)	Time (min)	Yield (%)	Recovery of IL (%)
1	[bmim][Cl]	100	120	32^a^	99
2	[bmim][BF_4_]	120	60	50^b^	46^c^
3	[bmim][NTf_2_]	120	60	61^b^	97^c^
4	[TMPA][NTf_2_]	100	120	57	88
5	[PP13][NTf_2_]	100	120	60	90
6	[DEME][NTf_2_]	100	120	32	92
7	[bmim][Cl]	100	120	35	97
8	[bmim][Cl]	100	120	83^d^	99

a Conventional heating was employed instead of microwave irradiation.

b Product partially contaminated with IL.

c IL partially contaminated with product 3a.

d Freshly prepared hydrazone 1a was used. When the reaction was carried out in the presence of 10 mol% of *N*,*N*-dimethylhydrazine, the yield of 3a dropped to less than 10%.

**Scheme 1:**
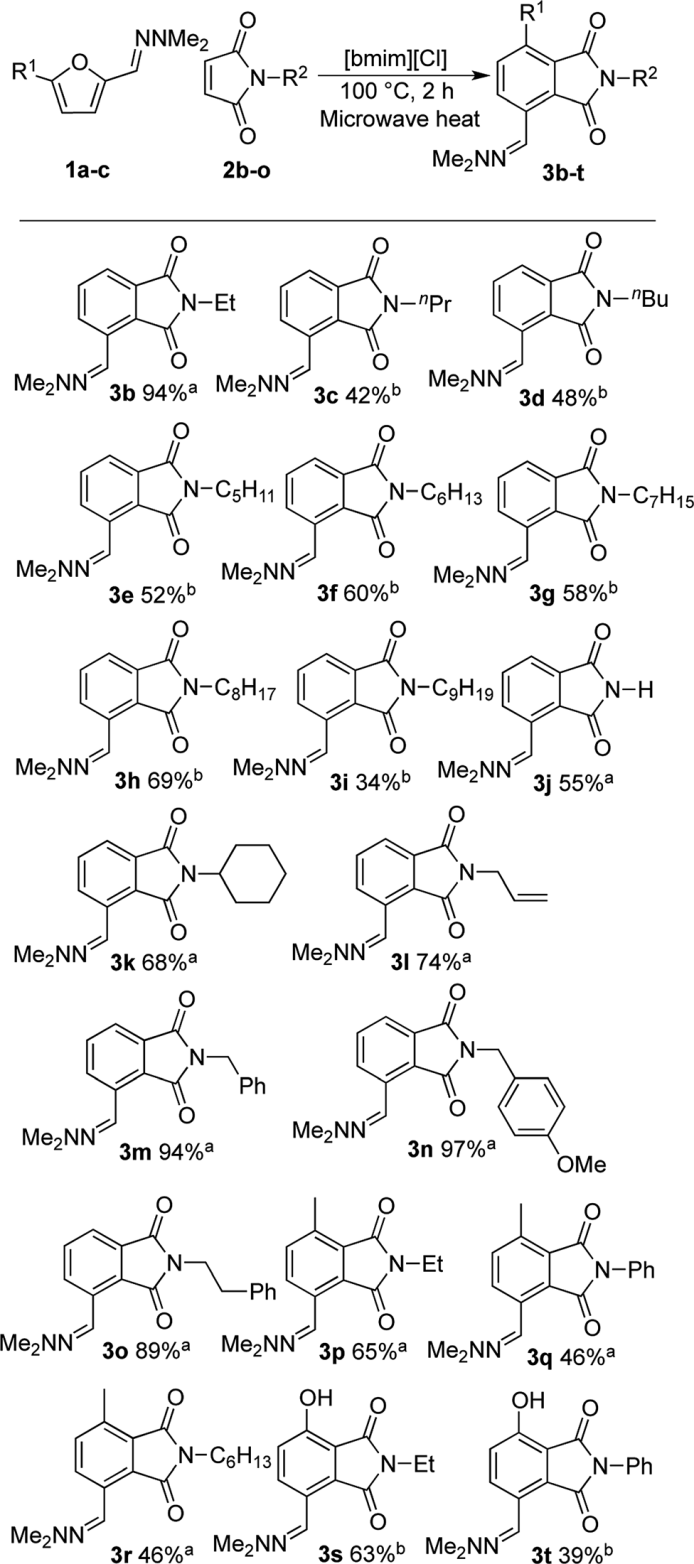
Cycloaddition products (3b**–**3t) were synthesised in a microwave reactor at 100 °C in 2 h with 1 g [bmim][Cl] as the solvent unless otherwise stated. The recovery of ionic liquid is >95% unless otherwise stated; ^a^ 1 g of IL used for 0.7 mmol of hydrazone and 1.5 eq. maleimide; ^b^ 2 g of IL used for 0.5 mmol of hydrazone and 1.0 eq. maleimide.

With satisfactory reaction conditions shown in Table 1, entry 8, we examined the applicability of the reaction conditions to a variety of furfural hydrazone derivatives 1 and maleimides 2 (Scheme 1). The reaction gave good yields of phthalimide products with aliphatic-substituted maleimides (3b–3i), including many examples of highly hydrophobic compounds that are unsuitable for reactions in water. The Diels–Alder reaction between 1a and 2b in IL gave phthalidimide 3b in 94% yield along with a 96% recovery of [bmim][Cl]. Longer aliphatic substituents on the maleimide were also tolerated, giving phthalimides 3c–3i in moderate to good yields. The reaction with a bulky maleimide such as 2k also progressed smoothly to give *N*-cyclohexylphthalimide 3k in 68% yield. Substituted benzyl maleimides similarly afforded the products 3m and 3n in excellent yields (94% and 97%, respectively). Note that the *N*-unsubstituted maleimide gave the cycloaddition product 3j in a good yield, which opens up the opportunity to further functionalisation at the nitrogen atom. *N*-Allyl substituted maleimide 2l underwent the cycloaddition to give the product 3l in 74% yield. We also examined the cycloaddition reaction using 5-methylfurfural hydrazone 1b, giving 3p, 3q and 3r in reasonable yields.

Treatment of 5-bromofurfural hydrazone 1c with maleimide 2a and 2b resulted in the formation of phenol derivative 3s and 3t, respectively. During the formation of these compounds, elimination of hydrogen bromide instead of dehydration occurred to aromatise the adduct. Unfortunately, the yield of 3t remained low because of its water solubility which caused loss of the product during the extraction/washing procedure.

To investigate the recyclability of the ionic liquids, we examined the reaction between furfural hydrazone 1a and *N*-ethylmaleimide 2b further (Table 2).

**Table 2:** Iterative use of [bmim][Cl]

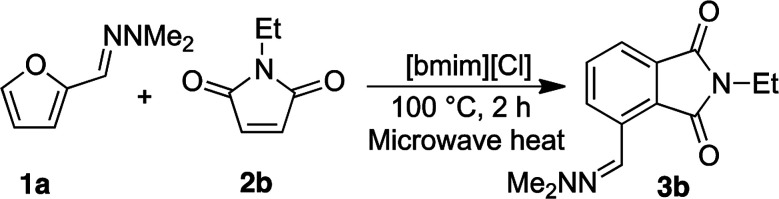		
Run	Yield (%)	[bmim][Cl] rec. (%)
1	94	100
2	92	100
3	92	96
4	66	88
5	87	97

The yields of 3b remained high in each of the five experiments, and an excellent recovery of [bmim][Cl] was achieved each time. ^1^H NMR spectroscopic data of the recovered IL indicated that the compound was identical to unused IL. These results clearly highlighted that [bmim][Cl] is stable under the reaction conditions used. Thus, the ionic liquids are useful for this conversion, and the efficient recovery and reuse of the ionic liquid is possible under these reaction conditions. Overall, a green conversion of furfurals into phthalimides using ionic liquids has been achieved.

## Conclusions

We have developed a successful protocol employing recyclable ionic liquids for the synthesis of substituted phthalimides from furfurals. The reaction proceeds efficiently under microwave heating with products isolated *via* a simple work-up procedure requiring a minimal amount of solvent to recover the ionic liquid. The products were recovered by recrystallisation from an acetone/water mixture without the need for chromatographic purification. The recyclability of the ionic liquid was >95% with no loss in efficiency over five cycles of use in the reaction. With such straightforward manipulations and the use of ionic liquids as a green solvent, the present protocol will contribute to the development of green chemical syntheses.

## Experimental section

### General methods

All reagents and solvents were purchased and used as supplied unless otherwise stated. All reactions were carried out under the atmosphere of nitrogen unless otherwise stated. All reactions were monitored by thin layer chromatography (TLC) or ^1^H NMR spectroscopy. TLC plates used were pre-coated with silica gel 60 F254 on aluminium (Merck KGaA) and visualised by UV light (254 nm) or chemically stained (KMnO_4_). Flash column chromatographic purification was performed using silica gel (Wako Co. Wakosil C-300). ^1^H NMR and ^13^C NMR spectra were recorded at 500 MHz (for ^1^H) 125 MHz on JEOL lamda-500 or JNM-ECA 500 Delta2 at ambient temperature, unless otherwise indicated. Deuterated solvents used for NMR spectroscopic characterisation were CDCl_3_, CD_3_OD, or DMSO-*d*_6_, as stated. Peaks are assigned as singlet (s), doublet (d), triplet (t), quintet (qn), sextet (sx), septet (sept), or multiplet (m). ^1^H and ^13^C shifts are reported in parts per million (ppm) and compared against residual solvent signals: CDCl_3_ (*δ* = 7.26 ppm, s; 77.2 ppm, t), CD_3_OD (*δ*_H_ 4.87, s and 3.31, qn; *δ*_C_ 49.1 ppm, sept), DMSO-*d*_6_ (*δ*_H_ 2.56 ppm, qn; *δ*_C_ 39.5 ppm, sept); ^1^H and ^13^C NMR shifts relative to TMS were calibrated using residual solvents peak. Infra-red spectra were obtained using a SHIMADZU FTIR-8400S spectrometer, all frequencies were given in reciprocal centimetres (cm^−1^). Melting points were measured on a YANACO MP-J3. High-resolution mass spectra were recorded on a JEOL JMS-T100LP mass spectrometer. Microwave heating was carried out using a SMW-087 microwave reactor, made by Shikoku Instrument Co. Ltd., 2.45 GHz, output 700 W, multimode. The reaction vessel (a 50 mL glass flask) was placed in the microwave oven (temperature was set at 100 °C or 120 °C). The reaction temperature was monitored by a thermocouple inserted into the reaction mixture and the reaction was stirred under microwave irradiation. Hydrazones 1 were prepared as described in the ESI.†

#### 4-((2,2-Dimethylhydrazono)methyl)-2-phenylisoindoline-1,3-dione 3a

A mixture of hydrazone 1a (96 mg, 0.70 mmol) and *N*-phenylmaleimide (182 mg, 1.05 mmol) in [bmim][Cl] (1.0324 g) was heated at 100 °C for 2 h in a microwave. Water (20 mL) was added and the reaction mixture was extracted with EtOAc (3 × 20 mL). The combined organic extracts were washed with water, brine, dried (Na_2_SO_4_), filtered, and concentrated *in vacuo*. The residue was recrystallised from acetone and cold water to obtain the product as a yellow solid (170 mg, 83%). The remaining water phase was concentrated and dried to recover the ionic liquid (1.0237 g, 99% recovery).

Yellow solid; mp 221–222 °C; ^1^H NMR (500 MHz, CDCl_3_) *δ* 8.31 (d, *J* = 8.1 Hz, 1H, NNCHCCH), 8.15 (s, 1H, NNCH), 7.72 (d, *J* = 7.2 Hz, 1H, NCHCCHCHCH), 7.64 (t, *J* = 7.8 Hz, 1H, NCHCCHCH), 7.53–7.46 (m, 2H, Ph), 7.44–7.37 (m, 3H, Ph), 3.13 (s, 6H, 2 × CH_3_); ^13^C NMR (125 MHz, CDCl_3_) *δ* 168.5, 167.5, 137.0, 133.8, 132.1, 131.8, 129.4, 129.2, 128.1, 126.8, 124.3, 124.1, 121.3, 42.7; HMRS: found (ESI): [M + H]^+^ 294.12390 C_17_H_16_N_3_O_2_, requires 294.12425; data in agreement with the literature.^4^

#### 4-((2,2-Dimethylhydrazono)methyl)-2-ethylisoindoline-1,3-dione 3b

A mixture of hydrazone 1a (96 mg, 0.70 mmol) and *N*-ethylmaleimide (131 mg, 1.05 mmol) in [bmim][Cl] (1.0291 g) was heated at 100 °C by microwave irradiation for 2 h. Water (20 mL) was added and the reaction mixture was extracted with EtOAc (3 × 15 mL). The combined organic extracts were washed with water, brine, dried (Na_2_SO_4_), filtered, and concentrated *in vacuo*. The residue was further recrystallised from acetone and cold water and the product collected by suction-filtration as a yellow solid (161 mg, 94%). The remaining water phase was concentrated and dried to recover the ionic liquid (1.0254 g, 100% recovery).

Yellow solid; mp 141–143 °C [lit.^4^ 142–143 °C]; ^1^H NMR (500 MHz, CDCl_3_) *δ* 8.22 (d, *J* = 7.7 Hz, 1H, N=CHCCH), 8.11 (s, 1H, N=CH), 7.61 (d, *J* = 7.7 Hz, 1H, N=CHCCHCHCH), 7.55 (t, *J* = 7.7 Hz, 1H, N=CHCCHCH), 3.72 (q, *J* = 7.3 Hz, 2H, CH_3_CH_2_), 3.12 (s, 6H, 2 × CH_3_), 1.26 (t, *J* = 7.3 Hz, 3H, CH_3_CH_2_); ^13^C NMR (125 MHz, CDCl_3_) *δ* 169.5, 168.5, 136.2, 133.4, 132.6, 129.0, 124.9, 124.7, 120.9, 42.7, 32.9, 14.1; HMRS: found (ESI): [M + H]^+^ 268.1068 C_13_H_15_N_3_NaO_2_, requires 268.1062; data in agreement with the literature.^4^

#### 4-((2,2-Dimethylhydrazino)methyl)-2-propylisoindoline-1,3-dione 3c

A mixture of hydrazone 1a (55 mg, 0.40 mmol) and *N*-propylmaleimide (112 mg, 0.81 mmol) in [bmim][Cl] (1.8394 g) was heated at 100 °C by microwave irradiation for 2 h. Water (10 mL) was added to the reaction mixture and the resulting mixture was extracted with EtOAc (5 × 10 mL). The combined organic extracts were washed with water, brine, dried (Na_2_SO_4_), filtered, and concentrated *in vacuo*. The residue was recrystallised from acetone and cold water and the product collected by suction-filtration as a yellow solid (75 mg, 73%). The remaining water phase was concentrated and dried to recover the ionic liquid (1.7979 g, 98% recovery).

Yellow solid; mp 121–122 °C; *ν*_max_ (solid per cm) 2964, 1703, 1552; ^1^H NMR (500 MHz, CDCl_3_) *δ* 8.20 (d, *J* = 8.0 Hz, 1H, NNCHCCH), 8.10 (s, 1H, NNCH), 7.59 (d, *J* = 7.2 Hz, 1H, NNCCHCHCH), 7.54 (t, *J* = 7.6 Hz, 1H, NNCHCCHCH), 3.61 (t, *J* = 7.3 Hz, 2H, NCH_2_CH_2_CH_3_), 3.11 (s, 6H, 2 × CH_3_), 1.72–1.65 (m, 2H, NCH_2_CH_2_CH_3_), 0.94 (t, *J* = 7.3 Hz, 3H, NCH_2_CH_2_CH_3_); ^13^C NMR (125 MHz, CDCl_3_) *δ* 169.7, 168.7, 136.3, 133.3, 132.4, 128.9, 124.7, 124.6, 120.8, 42.7, 39.5, 22.1, 11.5; HRMS: found (ESI): [M + H]^+^ 260.13883 C_14_H_18_N_3_O_2_, requires 260.13990.

#### 4-((2,2-Dimethylhydrazino)methyl)-2-butylisoindoline-1,3-dione 3d

A mixture of hydrazone 1a (96 mg, 0.69 mmol) and *N*-butylmaleimide (103 mg, 0.67 mmol) in [bmim][Cl] (2.01 g) was heated at 100 °C by microwave irradiation for 2 h. Water (10 mL) was added to the reaction mixture and the resulting mixture was extracted with EtOAc (5 × 10 mL). The combined organic extracts were washed with water, brine, dried (Na_2_SO_4_), filtered, and concentrated *in vacuo*. The residue was recrystallised from acetone and cold water and the product collected by suction-filtration as a yellow solid (88 mg, 48%). The remaining water phase was concentrated and dried to recover the ionic liquid (1.85 g, 92% recovery).

Yellow solid; mp 91–92 °C; ^1^H NMR (500 MHz, CDCl_3_) *δ* 8.22 (d, *J* = 8.0 Hz, 1H, NNCHCCH), 8.12 (s, 1H, NNCH), 7.61 (d, *J* = 7.2 Hz, 1H, NNCCHCHCH), 7.59–7.50 (m, 1H, NNCCHCH), 3.66 (t, *J* = 7.3 Hz, 2H, NCH_2_CH_2_CH_2_CH_3_), 3.12 (s, 6H, 2 × CH_3_), 1.68–1.62 (m, 2H, NCH_2_CH_2_CH_2_CH_3_), 1.38–1.32 (m, 2H, NCH_2_CH_2_CH_2_CH_3_), 0.94 (t, *J* = 7.4 Hz, 3H, NCH_2_CH_2_CH_2_CH_3_); ^13^C NMR (125 MHz, CDCl_3_) *δ* 169.6, 168.6, 136.3, 133.3, 132.4, 128.9, 124.8, 124.6, 120.8, 42.7, 37.7, 31.1, 20.2, 13.8; HRMS: found (ESI): [M + H]^+^ 274.15652 C_15_H_20_N_3_O_2_, requires 274.15555.

#### 4-((2,2-Dimethylhydrazino)methyl)-2-pentylisoindoline-1,3-dione 3e

A mixture of hydrazone 1a (73 mg, 0.53 mmol) and *N*-pentylmaleimide (102 mg, 0.61 mmol) in [bmim][Cl] (1.99 g) was heated at 100 °C by microwave irradiation for 2 h. Water (10 mL) was added to the reaction mixture and the resulting mixture was extracted with EtOAc (5 × 10 mL). The combined organic extracts were washed with water, brine, dried (Na_2_SO_4_), filtered, and concentrated *in vacuo*. The residue was recrystallised from acetone and cold water and the product collected by suction-filtration as a yellow solid (79 mg, 52%). The remaining water phase was concentrated and dried to recover the ionic liquid (1.95 g, 92% recovery).

Yellow solid; mp 74–75 °C; *ν*_max_ (solid per cm) 2995, 1697, 1548; ^1^H NMR (500 MHz, CDCl_3_) *δ* 8.20 (d, *J* = 8.0 Hz, 1H, NNCHCCH), 8.09 (s, 1H, NNCH), 7.61–7.56 (m, 1H, NNCCHCHCH), 7.53 (t, *J* = 7.7 Hz, 1H, NNCCHCH), 3.66–3.58 (m, 2H, NCH_2_CH_2_CH_2_CH_2_CH_3_), 3.10 (s, 6H, 2 × CH_3_), 1.68–1.60 (m, 2H, NCH_2_CH_2_CH_2_CH_2_CH_3_), 1.37–1.29 (m, 4H, NCH_2_ CH_2_CH_2_CH_2_CH_3_), 0.87 (t, *J* = 7.1 Hz, 3H, NCH_2_CH_2_CH_2_CH_2_CH_3_); ^13^C NMR (125 MHz, CDCl_3_) *δ* 169.6, 168.6, 136.3, 133.3, 132.4, 128.9, 124.8, 124.6, 120.8, 42.7, 38.0, 29.1, 28.4, 22.4, 14.1; HRMS: found (ESI): [M + H]^+^ 288.17265 C_16_H_22_N_3_O_2_, requires 288.17120.

#### 4-((2,2-Dimethylhydrazino)methyl)-2-hexylisoindoline-1,3-dione 3f

A mixture of hydrazone 1a (67.3 mg, 0.488 mmol) and *N*-hexylmaleimide (129.0 mg, 0.705 mmol) in [bmim][Cl] (1.083 g) was heated at 100 °C by microwave irradiation for 2 h. Water (10 mL) was added to the reaction mixture and the resulting mixture was extracted with EtOAc (5 × 10 mL). The combined organic extracts were washed with water, brine, dried (Na_2_SO_4_), filtered, and concentrated *in vacuo*. The residue was recrystallised from acetone and cold water and the product collected by suction-filtration as a yellow solid (88.7 mg, 60%). The remaining water phase was concentrated and dried to recover the ionic liquid (1.029 g, 95% recovery).

Yellow solid; mp 73–74 °C; *ν*_max_ (solid per cm) 2929, 1699, 1550; ^1^H NMR (500 MHz, CDCl_3_) *δ* 8.18 (d, *J* = 7.8 Hz, 1H, NNCHCCH), 8.08 (s, 1H, NN=CH), 7.57 (d, *J* = 8.2 Hz, 1H, NNCCHCHCH), 7.52 (t, *J* = 7.6 Hz, 1H, NNCCHCH), 3.62 (d, *J* = 7.4 Hz, 2H, NCH_2_CH_2_CH_2_CH_2_CH_2_CH_3_), 3.09 (s, 6H, 2 × CH_3_), 1.63 (p, *J* = 7.4 Hz, 2H, NCH_2_CH_2_CH_2_CH_2_CH_2_CH_3_), 1.36–1.13 (m, 6H, NCH_2_ CH_2_CH_2_CH_2_CH_2_CH_3_), 0.85 (t, *J* = 6.9 Hz, 3H, N–(CH_2_)_5_–CH_3_); ^13^C NMR (125 MHz, CDCl_3_) *δ* 169.6, 168.6, 136.3, 133.3, 132.4, 128.9, 124.8, 124.6, 120.8, 42.7, 38.0, 31.5, 28.7, 26.7, 22.6, 14.1; HRMS: found (ESI): [M + Na]^+^ 324.16711 C_17_H_23_N_3_O_2_Na, requires 324.16880.

#### 4-((2,2-Dimethylhydrazino)methyl)-2-heptylisoindoline-1,3-dione 3g

A mixture of hydrazone 1a (76 mg, 0.55 mmol) and *N*-heptylmaleimide (105 mg, 0.54 mmol) in [bmim][Cl] (1.98 g) was heated at 100 °C by microwave irradiation for 2 h. Water (10 mL) was added to the reaction mixture and the resulting mixture was extracted with EtOAc (5 × 10 mL). The combined organic extracts were washed with water, brine, dried (Na_2_SO_4_), filtered, and concentrated *in vacuo*. The residue was recrystallised from acetone and cold water and the product collected by suction-filtration as a yellow solid (99 mg, 58%). The remaining water phase was concentrated and dried to recover the ionic liquid (1.94 g, 98% recovery).

Yellow solid; mp 55–56 °C; *ν*_max_ (solid per cm) 2928, 1699, 1550; ^1^H NMR (500 MHz, CDCl_3_) *δ* 8.21 (d, *J* = 8.0 Hz, 1H, NNCHCCH), 8.12 (s, 1H, NNCH), 7.60 (d, *J* = 7.3 Hz, 1H, NNCHCCHCHCH), 7.56 (d, *J* = 7.6 Hz, 1H, NNCHCCHCH), 3.68–3.60 (m, 2H, NCH_2_(CH_2_)_5_CH_3_), 3.11 (s, 6H, 2 × CH_3_), 1.70–1.61 (m, 2H, NCH_2_CH_2_(CH_2_)_4_CH_3_), 1.36–1.29 (m, 4H, 2 × CH_2_), 1.29–1.18 (m, 4H, 2 × CH_2_), 0.86 (t, *J* = 6.8 Hz, 3H, N(CH_2_)_6_CH_3_); ^13^C NMR (125 MHz, CDCl_3_) *δ* 169.4, 168.4, 136.2, 133.1, 132.4, 128.7, 124.6, 124.4, 120.6, 42.5, 37.9, 31.8, 28.9, 28.7, 26.9, 22.6, 14.1.; HRMS: found (ESI): [M + H]^+^ 316.20250 C_18_H_26_N_3_O_2_, requires 316.20250.

#### 4-((2,2-Dimethylhydrazino)methyl)-2-octylisoindoline-1,3-dione 3h

A mixture of hydrazone 1a (99.7 mg, 0.72 mmol) and *N*-octylmaleimide (210 mg, 1.00 mmol) in [bmim][Cl] (1.012 g) was heated at 100 °C by microwave irradiation for 2 h. Water (10 mL) was added to the reaction mixture and the resulting mixture was extracted with EtOAc (5 × 10 mL). The combined organic extracts were washed with water, brine, dried (Na_2_SO_4_), filtered, and concentrated *in vacuo*. The residue was recrystallised from acetone and cold water and the product collected by suction-filtration as a yellow solid (119.3 mg, 50%). The remaining water phase was concentrated and dried to recover the ionic liquid (0.958 g, 95% recovery).

Yellow solid; mp 50–51 °C; *ν*_max_ (solid per cm) 2926, 1703, 1550; ^1^H NMR (500 MHz, CDCl_3_) *δ* 8.21 (d, *J* = 7.9 Hz, 1H), 8.11 (s, 1H), 7.60 (d, *J* = 7.1 Hz, 1H), 7.54 (t, *J* = 7.6 Hz, 1H), 3.63 (t, *J* = 7.3 Hz, 2H), 3.11 (s, 6H), 1.65 (m, *J* = 7.3 Hz, 2H), 1.34–1.22 (m, 10H), 0.85 (t, *J* = 6.7 Hz, 3H); ^13^C NMR (125 MHz, CDCl_3_) *δ* 169.6, 168.6, 136.3, 133.3, 132.5, 128.9, 124.8, 124.7, 120.8, 42.7, 38.0, 31.9, 29.3, 29.3, 28.8, 27.0, 22.7, 14.2; HRMS: found (ESI): [M + Na]^+^ 352.19881 C_19_H_27_N_3_O_2_Na, requires 352.20010.

#### 4-((2,2-Dimethylhydrazino)methyl)-2-nonylisoindoline-1,3-dione 3i

A mixture of hydrazone 1a (73 mg, 0.53 mmol) and *N*-nonylmaleimide (118 mg, 0.53 mmol) in [bmim][Cl] (2.00 g) was heated at 100 °C by microwave irradiation for 2 h. Water (10 mL) was added to the reaction mixture and the resulting mixture was extracted with EtOAc (5 × 10 mL). The combined organic extracts were washed with water, brine, dried (Na_2_SO_4_), filtered, and concentrated *in vacuo*. The residue was recrystallised from acetone and cold water and the product collected by suction-filtration as a yellow solid (62 mg, 34%). The remaining water phase was concentrated and dried to recover the ionic liquid (1.98 g, 99% recovery).

Yellow solid; mp 42–43 °C; *ν*_max_ (solid per cm) 2924, 1701, 1550; ^1^H NMR (500 MHz, CDCl_3_) *δ* 8.21 (d, *J* = 8.0 Hz, 1H, NNCHCCH), 8.11 (s, 1H, NNCH), 7.60 (d, *J* = 7.2 Hz, 1H, NNCHCCHCHCH), 7.55 (t, *J* = 7.6 Hz, 1H, NNCHCCHCH), 3.64 (t, *J* = 7.3 Hz, 2H, NCH_2_(CH_2_)_8_CH_3_), 3.11 (s, 6H, 2 × CH_3_), 1.66–1.62 (m, 2H, NCH_2_CH_2_(CH_2_)_6_CH_3_), 1.37–1.22 (m, 4H, 2 × CH_2_), 1.27–1.22 (m, 8H, 4 × CH_2_), 0.86 (t, *J* = 6.9 Hz, 3H, NCH_2_(CH_2_)_8_CH_3_); ^13^C NMR (125 MHz, CDCl_3_) *δ* 169.6, 168.6, 136.3, 134.1, 133.3, 132.4, 128.9, 124.6, 120.8, 42.7, 38.0, 31.9, 29.5, 29.3, 29.2, 28.8, 27.0, 22.8, 14.2; HRMS: found (ESI): [M + Na]^+^ 366.21742 C_20_H_29_N_3_O_2_Na, requires 366.21575.

#### 4-((2,2-Dimethylhydrazono)methyl)isoindoline-1,3-dione 3j

A mixture of hydrazone 3a (96 mg, 0.70 mmol) and maleimide (103 mg, 1.05 mmol) in [bmim][Cl] (1.0085 g) was heated at 100 °C by microwave irradiation for 2 h. Water (20 mL) was added and the reaction mixture was extracted with EtOAc (4 × 20 mL). The combined organic extracts were washed with water, brine, dried (Na_2_SO_4_), filtered, and concentrated *in vacuo*. The residue was recrystallised from acetone and cold water and the product collected by suction-filtration as a yellow solid (81 mg, 53%). The remaining water phase was concentrated and dried to recover the ionic liquid (0.9180 g, 91% recovery).

Yellow solid; mp 240–241 °C; ^1^H NMR (500 MHz, CDCl_3_) *δ* 8.27 (d, *J* = 7.2 Hz, 1H, NNCHCCH), 8.06 (s, 1H, NCH), 7.63–7.58 (m, 3H, NNCHCCHCH, NNCHCCHCHCH and N–H), 3.13 (s, 6H, 2 × CH_3_); ^13^C NMR (125 MHz, CDCl_3_) *δ* 169.0, 168.0, 137.0, 133.8, 133.0, 129.3, 125.1, 124.3, 121.2, 42.8; HRMS found (ESI): [M + Na]^+^ 240.0743 C_11_H_11_N_3_NaO_2_, requires 240.0749.

#### 2-Cyclohexyl-4-((2,2-dimethylhydrazono)methyl)isoindoline-1,3-dione 3k

A mixture of hydrazone 1a (45 mg, 0.33 mmol, 1.0 eq.) and *N*-cyclohexylmaleimide (88 mg, 0.49 mmol, 1.5 eq.) in [bmim][Cl] (0.517 g) was heated at 100 °C by microwave irradiation for 2 h. Water (10 mL) was added to the reaction mixture and the resulting mixture was extracted with EtOAc (3 × 10 mL). The combined organic phase was washed with brine, dried over Na_2_SO_4_ and filtered. The filtrate was concentrated to give a crude product which was recrystallised from water-acetone to give 3k as a yellow solid (67 mg, 68%). The remaining water phase was concentrated and dried to recover the ionic liquid (0.509 g, 98% recovery).

Yellow solid; mp 187–189 °C; *ν*_max_ (solid per cm) 2951, 2918, 2851, 1755, 1697, 1549, 1464; ^1^H NMR (500 MHz, CDCl_3_) *δ* 8.20 (d, *J* = 7.5 Hz, 1H, NNCHCCH), 8.12 (s, 1H, NCH), 7.58 (d, *J* = 7.5 Hz, 1H, NNCCHCHCH), 7.54 (t, *J* = 7.5 Hz, 1H, NNCCHCH), 4.13–4.03 (m, 1H, CONCH), 3.11 (s, 6H, 2 × CH_3_), 2.25–2.13 (m, 2H, CyCH_2_), 1.87–1.84 (m, 2H, CyCH_2_), 1.73–1.71 (m, 2H, CyCH_2_), 1.67 (s, 1H, CyCH_2_), 1.43–1.17 (m, 2H, CyCH_2_), 0.98–0.74 (m, 1H, CyCH_2_); ^13^C NMR (125 MHz, CDCl_3_) *δ* 169.8, 168.6, 136.1, 133.2, 132.3, 128.9, 124.7, 120.7, 50.8, 42.7, 30.0, 29.8, 26.2, 25.3; HRMS: found (ESI): [M + H]^+^ 300.1727 C_17_H_22_N_3_O_2_, requires 300.1707.

#### 2-Allyl-4-((2,2-dimethylhydrazono)methyl)isoindoline-1,3-dione 3l

A mixture of hydrazone 1a (101 mg, 0.73 mmol) and *N*-allylmaleimide (147 mg, 1.05 mmol) in [bmim][Cl] (1.0070 g) was heated at 100 °C by microwave irradiation for 2 h. Water (20 mL) was added and the reaction mixture was extracted with EtOAc (5 × 10 mL). The combined organic extracts were washed with water, brine, dried (Na_2_SO_4_), filtered, and concentrated *in vacuo*. The residue was recrystallised from acetone and cold water and the product collected by suction-filtration as a yellow solid (175 mg, 93%). The remaining water phase was concentrated and dried to recover the ionic liquid (0.9768 g, 97% recovery).

Yellow solid; mp 128–129 °C; *ν*_max_ (solid per cm) 2986, 2924, 2889, 2359, 2332, 1759, 1701, 1547, 1427; ^1^H NMR (500 MHz, CDCl_3_) *δ* 8.22 (d, *J* = 8.0 Hz, 1H, NNCHCCH), 8.08 (s, 1H, NNCH), 7.61 (d, *J* = 7.2 Hz, 1H, NNCHCCHCHCH), 7.55 (t, *J* = 7.7 Hz, 1H, NNCHCCHCH), 5.94–5.82 (m, 1H, CHH=CH), 5.30–5.21 (m, 1H, CHH=CH), 5.18 (d, *J* = 10.2 Hz, 1H, CH_2_=CH), 4.26 (d, *J* = 5.5 Hz, 2H, NCH_2_), 3.11 (s, 6H, 2 × CH_3_); ^13^C NMR (125 MHz, CDCl_3_) *δ* 169.1, 168.1, 136.5, 133.4, 132.4, 131.9, 129.0, 124.6, 124.4, 121.0, 117.6, 42.7, 40.0; HRMS: found (ESI): [M + H]^+^ 258.1256 C_14_H_16_N_3_O_2_, requires 258.1237.

#### 2-Benzyl-4-((2,2-dimethylhydrazono)methyl)isoindoline-1,3-dione 3m

A mixture of hydrazone 1a (100 mg, 0.70 mmol, 1.0 eq.) and *N*-benzylmaleimide (198 mg, 1.05 mmol, 1.5 eq.) in [bmim][Cl] (1.0015 g) was heated at 100 °C by microwave irradiation for 2 h. Water (10 mL) was added to the reaction mixture and the resulting mixture was extracted with EtOAc (4 × 10 mL). The combined organic phase was washed with brine, dried over Na_2_SO_4_ and filtered. The filtrate was concentrated to give a crude product which was recrystallised from water–acetone to give 5c as a yellow solid (208 mg, 94%). The remaining water phase was concentrated and dried to recover the ionic liquid (0.9633 g, 96% recovery).

Yellow solid; mp 48–49 °C; ^1^H NMR (500 MHz, CDCl_3_) *δ* 8.22 (d, *J* = 8.0 Hz, 1H, NNCHCCH), 8.08 (s, 1H, NNCH), 7.61 (d, *J* = 7.0 Hz, 1H, NNCHCCHCHCH), 7.55 (t, *J* = 7.7 Hz, 1H, NNCHCCHCH), 7.42 (d, *J* = 7.3 Hz, 2H, Ph), 7.35–7.22 (m, 3H, Ph), 4.82 (s, 2H, PhCH_2_), 3.11 (s, 6H, 2 × CH_3_); ^13^C NMR (125 MHz, CDCl_3_) *δ* 169.2, 168.3, 136.7, 136.5, 133.4, 132.4, 129.1, 128.8, 128.6, 127.8, 124.7, 124.4, 121.0, 42.7, 41.5; HRMS: found (ESI): [M + H]^+^ 308.14024 C_18_H_18_N_3_O_2_, requires 308.13990; data in agreement with the literature.^4^

#### 4-((2,2-Dimethylhydrazono)methyl)-2-(4-methoxybenzyl)-isoindoline-1,3-dione 3n

A mixture of hydrazone 1a (100 mg, 0.72 mmol) and *N*-4-methoxybenzylmaleimide (230 mg, 1.05 mmol) in [bmim][Cl] (0.9541 g) was heated at 100 °C by microwave irradiation for 2 h. Water (20 mL) was added and the reaction mixture was extracted with EtOAc (5 × 10 mL). The combined organic extracts were washed with water, brine, dried (Na_2_SO_4_), filtered, and concentrated *in vacuo*. The residue was recrystallised from acetone and cold water and the product collected by suction-filtration as a yellow solid (229 mg, 93%). The remaining water phase was concentrated and dried to recover the ionic liquid (0.9395 g, 98% recovery).

Yellow solid; mp 129–131 °C; *ν*_max_ (solid per cm) 2949, 2833, 1755, 1697, 1611, 1549, 1510; ^1^H NMR (500 MHz, CDCl_3_) *δ* 8.21 (d, *J* = 8.0 Hz, 1H, NNCHCCH), 8.08 (s, 1H, NNCH), 7.63–7.57 (m, 1H, NNCHCCHCHCH), 7.54 (t, *J* = 7.6 Hz, 1H, NNCHCCHCH), 7.37 (d, *J* = 8.6 Hz, 2H, Ar), 6.86–6.80 (m, 2H, Ar), 4.76 (s, 2H, ArCH_2_), 3.76 (s, 3H, OCH_3_), 3.11 (s, 6H, 2 × CH_3_); ^13^C NMR (125 MHz, CDCl_3_) *δ* 169.2, 168.3, 159.2, 136.5, 133.4, 132.4, 130.1, 129.0, 128.9, 124.7, 124.5, 121.0, 114.1, 55.4, 42.7, 41.0; HRMS: found (ESI): [M + H]^+^ 338.15041 C_19_H_20_N_3_O_3_, requires 338.15047.

#### 4-((2,2-Dimethylhydrazono)methyl)-2-phenethylisoindoline-1,3-dione 3o

A mixture of hydrazone 1a (100 mg, 0.72 mmol) and *N*-phenethylmaleimide (145 mg, 1.05 mmol) in [bmim][Cl] (1.0348 g) was heated at 100 °C by microwave irradiation for 2 h. Water (20 mL) was added and the reaction mixture was extracted with EtOAc (3 × 20 mL). The combined organic extracts were washed with water, brine, dried (Na_2_SO_4_), filtered, and concentrated *in vacuo*. The residue was recrystallised from acetone and cold water and the product collected by suction-filtration as a yellow solid (208 mg, 89%). The remaining water phase was concentrated and dried to recover the ionic liquid (1.0327 g, >99% recovery).

Yellow solid; mp 106–107 °C; *ν*_max_ (solid per cm) 2931, 2864, 1763, 1694, 1541, 1454; ^1^H NMR (500 MHz, CDCl_3_) *δ* 8.22 (d, *J* = 7.9 Hz, 1H, Ar), 8.09 (s, 1H, NCH), 7.62–7.58 (m, 1H, Ar), 7.55 (t, *J* = 7.6 Hz, 1H, Ar), 7.31–7.25 (m, 4H, Ph), 7.24–7.19 (m, 1H, Ph), 3.94–3.83 (m, 2H, PhCH_2_CH_2_), 3.12 (s, 6H, 2 × CH_3_), 3.02–2.92 (m, 2H, PhCH_2_); ^13^C NMR (125 MHz, CDCl_3_) *δ* 169.4, 168.3, 138.2, 136.4, 133.4, 132.4, 129.0, 128.9, 128.7, 126.7, 124.7, 124.5, 120.9, 42.7, 39.3, 34.9; HRMS: found (ESI): [M + H]^+^ 322.15728 C_19_H_20_N_3_O_2_, requires 322.15555.

#### 4-((2,2-Dimethylhydrazono)methyl)-2-ethyl-7-methylisoindoline-1,3-dione 3p

A mixture of hydrazone 1b (111 mg, 0.73 mmol) and *N*-ethylmaleimide (139 mg, 1.11 mmol) in [bmim][Cl] (1.0145 g) was heated at 100 °C by microwave irradiation for 2 h. Water (20 mL) was added and the reaction mixture was extracted with EtOAc (5 × 10 mL). The combined organic extracts were washed with water, brine, dried (Na_2_SO_4_), filtered, and concentrated *in vacuo*. The residue was recrystallised from acetone and cold water and the product collected by suction-filtration as a yellow solid (119 mg, 65%). The remaining water phase was concentrated and dried to recover the ionic liquid (1.0013 g, 98% recovery).

Yellow solid; mp 142–143 °C; ^1^H NMR (500 MHz, CDCl_3_) *δ* 8.14 (s, 1H, NNCH), 8.08 (d, *J* = 8.3 Hz, 1H, NNCHCCH), 7.30 (d, *J* = 8.3 Hz, 1H, NNCHCCHCH), 3.68 (d, *J* = 7.1 Hz, 2H, CH_2_CH_3_), 3.08 (s, 6H, 2 × CH_3_), 2.63 (s, 3H, CH_3_), 1.24 (t, *J* = 7.1 Hz, 3H, CH_2_CH_3_);^13^C NMR (125 MHz, CDCl_3_) *δ* 169.3, 169.1, 136.2, 135.8, 134.1, 128.8, 128.7, 125.6, 125.2, 42.7, 32.6, 17.6, 14.1; HRMS: found (ESI): [M + H]^+^ 260.1398 C_14_H_18_N_3_O_3_, requires 260.1399; data in agreement with the literature.^4^

#### 4-((2,2-Dimethylhydrazono)methyl)-7-methyl-2-phenylisoindoline-1,3-dione 3q

A mixture of hydrazone 1b (119 mg, 0.78 mmol) and *N*-phenylmaleimide (197 mg, 1.14 mmol) in [bmim][Cl] (0.9931 g) was heated at 100 °C by microwave irradiation for 2 h. Water (20 mL) was added and the reaction mixture was extracted with EtOAc (5 × 10 mL). The combined organic extracts were washed with water, brine, dried (Na_2_SO_4_), filtered, and concentrated *in vacuo*. The residue was recrystallised from acetone and cold water and the product collected by suction-filtration as a yellow solid (109 mg, 46%). The remaining water phase was concentrated and dried to recover the ionic liquid (0.9893 g, 99% recovery).

Yellow solid; mp 201–203 °C; *ν*_max_ (solid per cm) 1753, 1694, 1541, 1489; ^1^H NMR (500 MHz, CDCl_3_) *δ* 8.18–8.17 (m, 2H, NNCH and NNCHCCH), 7.49 (t, *J* = 7.8 Hz, 2H, Ph), 7.45–7.36 (m, 4H, Ph and NNCHCCHCH), 3.09 (s, 6H, 2 × CH_3_), 2.69 (s, 3H, CH_3_);^13^C NMR (125 MHz, CDCl_3_) *δ* 168.4, 168.1, 136.7, 136.4, 134.7, 131.9, 129.2, 129.1, 128.2, 128.0, 126.9, 125.3, 124.5, 42.7, 17.8; HRMS: found (ESI): [M + H]^+^ 308.1341 C_18_H_28_N_3_O_3_, requires; 308.1399.

#### 4-((2,2-Dimethylhydrazono)methyl)-2-hexyl-7-methylisoindoline-1,3-dione 3r

A mixture of hydrazone 1b (80 mg, 0.69 mmol) and *N*-hexylmaleimide (154 mg, 0.84 mmol) in [bmim][Cl] (1.00 g) was heated at 100 °C by microwave irradiation for 2 h. Water (20 mL) was added and the reaction mixture was extracted with EtOAc (5 × 10 mL). The combined organic extracts were washed with water, brine, dried (Na_2_SO_4_), filtered, and concentrated *in vacuo*. The residue was recrystallised from acetone and cold water and the product collected by suction-filtration as a yellow solid (110 mg, 67%). The remaining water phase was concentrated and dried to recover the ionic liquid (0.97 g, 97% recovery).

Yellow solid; mp 80–81 °C; *ν*_max_ (solid per cm) 2929, 1693, 1548; ^1^H NMR (500 MHz, CDCl_3_) *δ* 8.13 (s, 1H, NNCH), 8.07 (d, *J* = 8.2 Hz, 1H, NNCHCCH), 7.29 (d, *J* = 8.2 Hz, 1H, NNCHCCHCH), 3.64–3.56 (m, 2H, NCH_2_(CH_2_)_4_CH_3_), 3.07 (s, 6H, 2 × CH_3_), 2.63 (s, 3H, CH_3_), 1.66–1.60 (m, 2H, CH_2_), 1.34–1.28 (m, 6H, 3 × CH_2_), 0.86 (t, *J* = 6.9 Hz, 3H, NCH_2_(CH_2_)_4_CH_3_);^13^C NMR (125 MHz, CDCl_3_) *δ* 169.5, 169.3, 136.2, 135.8, 134.0, 128.7, 128.6, 125.6, 125.1, 42.7, 37.8, 31.5, 28.7, 26.7, 22.6, 17.6, 14.1; HRMS: found (ESI): [M + H]^+^ 316.20474 C_18_H_26_N_3_O_2_, requires 316.20250.

#### 4-((2,2-Dimethylhydrazono)methyl)-2-ethyl-7-hydroxyisoindoline-1,3-dione 3s

A mixture of hydrazone 1c (72 mg, 0.35 mmol) and *N*-ethylmaleimide (67 mg, 0.53 mmol) in [bmim][Cl] (1.595 g) was heated at 100 °C by microwave irradiation for 2 h. Water (20 mL) was added and the reaction mixture was extracted with EtOAc (5 × 20 mL). The combined organic extracts were washed with water, brine, dried (Na_2_SO_4_), filtered, and concentrated *in vacuo*. The residue was recrystallised from acetone and cold water and the product collected by suction-filtration as a red-brown solid (58 mg, 63%). The remaining water phase was concentrated and dried to recover the ionic liquid (1.67 g, >100% recovery, partial contamination with product). Data in agreement with the literature.^4^

Red-brown solid; mp 147–148 °C; ^1^H NMR (500 MHz, CDCl_3_) *δ* 8.15 (d, *J* = 8.7 Hz, 1H, NNCHCCH), 8.00 (s, 1H, NNCH), 7.73 (br s, 1H, OH), 7.07 (d, *J* = 8.7 Hz, 1H, NNCHCCHCH), 3.69 (q, *J* = 7.3 Hz, 2H, CH_2_), 3.07 (s, 6H, 2 × CH_3_), 1.27 (t, *J* = 7.3 Hz, 3H, CH_3_); ^13^C NMR (125 MHz, CDCl_3_) *δ* 170.5, 168.6, 153.7, 132.4, 129.8, 125.3, 124.2, 123.1, 113.5, 42.8, 32.7, 14.2; HRMS: found (ESI): [M + Na]^+^ 284.1018 C_13_H_15_N_3_NaO_3_, requires 284.1011.

#### 4-((2,2-Dimethylhydrazono)methyl)-7-hydroxy-2-phenylisoindoline-1,3-dione 3t

A mixture of hydrazone 1c (50 mg, 0.25 mmol) and *N*-phenylmaleimide (52 mg, 0.30 mmol) in [bmim][Cl] (1.869 g) was heated at 100 °C by microwave irradiation for 2 h. Water (20 mL) was added and the reaction mixture was extracted with EtOAc (5 × 20 mL). The combined organic extracts were washed with water, brine, dried (Na_2_SO_4_), filtered, and concentrated *in vacuo*. The residue was recrystallised from acetone and cold water and the product collected by suction-filtration as a red-brown solid (29 mg, 39%). The remaining water phase was concentrated and dried to recover the ionic liquid (1.860 g, 99%).

Red-brown solid; mp 227–229 °C; *ν*_max_ (KBr/cm^−1^) 3246, 1763, 1686, 1557, 1493; ^1^H NMR (500 MHz, CDCl_3_) *δ* 8.24 (d, *J* = 8.8 Hz, 1H, NNCHCCH), 8.03 (s, 1H, NNCH), 7.88 (s, 1H, OH), 7.49 (d, *J* = 7.8 Hz, 2H, Ph), 7.46–7.34 (m, 3H, Ph), 7.15 (d, *J* = 8.8 Hz, 1H, NNCHCCHCH), 3.07 (s, 6H, 2 × CH_3_); ^13^C NMR (125 MHz, CDCl_3_) *δ* 169.5, 167.6, 154.2, 132.9, 131.4, 130.3, 129.2, 128.2, 126.5, 124.9, 123.6, 123.2, 113.0, 42.76; HRMS: found (ESI): [M + H]^+^ 301.1184 C_17_H_16_N_3_O_2_, requires 310.1192.

#### Recycling [bmim][Cl] in the synthesis of phthalimide 3b

A mixture of hydrazone 3a (1.0 eq.) and *N*-ethylmaleimide (1.5 eq.) in [bmim][Cl] (8.2 eq.) was heated at 100 °C by microwave irradiation for 2 h. Water (20 mL) was added and the reaction mixture was extracted with EtOAc (3 × 15 mL). The combined organic extracts were washed with water, brine, dried (Na_2_SO_4_), filtered, and concentrated *in vacuo*. The residue was further recrystallised from acetone with cold water and the product collected by suction-filtration as a yellow solid. The remaining water phase was concentrated to obtain recovered ionic liquid, which was then dissolved in CH_2_Cl_2_ and dried over Na_2_SO_4_. Filtration and concentration of the filtrate gave the recovered ionic liquid, which was used for the synthesis of 3b again. The same procedure was repeated five times.

## Conflicts of interest

There are no conflicts to declare.

## Supplementary Material

RA-008-C8RA03895C-s001
