# Supplementary material for: Development of a microwave-assisted sustainable conversion of furfural hydrazones to functionalised phthalimides in ionic liquids
Source: RSC Adv. 2018 Jun 20;8(40):22617–24. doi: 10.1039/c8ra03895c (PMC9081452; doi:10.1039/c8ra03895c)

Electronic Supplementary Material (ESI) for RSC Advances.
This journal is © The Royal Society of Chemistry 2018

Electronic Supplementary Information (ESI)

Development of a microwave-assisted sustainable conversion of furfural hydrazones to functionalised phthalimides in ionic liquids

Valerija Karaluka,[a†] Kengo Murata,[a†] Shinto Masuda,[a] Yuto Shiramatsu,[a] Takuji Kawamoto,[a] Helen C. Hailes,*[b] Tom D. Sheppard,*[b] and Akio Kamimura*[a]

a. *Department of Applied Molecular Biosciences, Graduate School of Medicine, Yamaguchi University, Ube 755-8611 Japan*
b. *Department of Chemistry, University College London, 20 Gordon Street, London, WC1H 0AJ.*

† *Authors contributed equally*

Email: ak10@yamaguchi-u.ac.jp

Contents

synthesis of hydrazone 2

NMR spectra 4

Synthesis of hydrazones 1

2-Furaldehyde dimethylhydrazone 1a

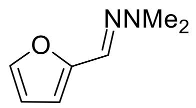

A mixture of furfural (0.83 mL, 10.0 mmol) and *N,N*-dimethylhydrazine (0.91 mL, 12.0 mmol) in EtOH (60 mL) was stirred at room temperature for 90 min. Water (30 mL) was added and the reaction mixture was concentrated using a rotary evaporator to remove EtOH. The remaining aqueous mixture was extracted with CH_2Cl_2 (3 × 30 mL). The organic layer was dried (Na_2SO_4), filtered and concentrated *in vacuo* to afford the product as a brown liquid (1.29 g, 94%); ν_{max} (film/cm^{-1}); ^{1}H NMR (500 MHz, $CDCl_3$) δ 7.37 (br s, 1H, OCH), 7.12 (s, 1H, NCH), 6.39 (dd, *J* = 3.3, 1.8 Hz, 1H, CCHC*H*), 6.35 (d, *J* = 3.3 Hz, 1H, CCH), 2.95 (s, 6H, 2 × CH_3); ^{13}C NMR (125 MHz, $CDCl_3$) δ 152.2, 142.0, 123.4, 111.3, 107.4, 42.9; HRMS: Found (ESI): $[M+H]^+$ 139.08729 $C_7H_{11}N_2O$, requires 139.08714; Data in agreement with the literature.[1]

5-Methyl-2-furaldehyde dimethylhydrazone 1b

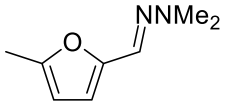

A mixture of 5-methyl-2-furfural (0.99 mL, 10.0 mmol) and *N,N*-dimethylhydrazine (0.83 mL, 11.0 mmol) in EtOH (200 mL) was stirred at room temperature for 3 h. Water (30 mL) was added and EtOH was removed *in vacuo*. The remaining aqueous mixture was extracted with Et_2O (3 × 50 mL). The organic layer was dried (Na_2SO_4), filtered and concentrated *in vacuo* to afford the product as an orange liquid (1.18 g, 76%); ^{1}H NMR (500 MHz, $CDCl_3$) δ 7.09 (s, 1H, NCH), 6.24 (d, *J* = 2.8 Hz, 1H, NCCH), 5.98 (d, *J* = 2.8 Hz, 1H, CH_3CC*H*), 2.92 (s, 6H, 2 × CH_3), 2.33 (s, 3H, CH_3); ^{13}C NMR (125 MHz, $CDCl_3$) δ 152.4, 150.5, 124.5, 109.3, 107.5, 77.37, 43.1, 13.9.

5-Bromo-2-furaldehyde dimethylhydrazone 1c

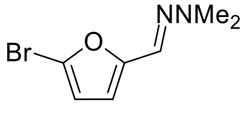

A mixture of 5-bromo-2-fulfural (352 mg, 2.01 mmol), $MgSO_4$ (808 mg), and *N,N*-dimethylhydrazine (176 mg, 2.94 mmol) in CH_2Cl_2 (2 mL) was stirred at room temperature for 15 h. The reaction mixture was filtered and the filtrate was concentrated to give crude hydrazone as a pale red liquid (407 mg, 99 %); ^{1}H NMR (500 MHz, $CDCl_3$) δ 6.99 (s, 1H,

NCH), 6.31 (d, J = 3.5 Hz, 1H, NCCH), 6.31 (d, J = 3.5 Hz, 1H, BrCCH), 2.95 (s, 6H, 2 × CH_3); ^{13}C NMR (125 MHz, $CDCl_3$) δ 154.2, 121.8, 121.1, 113.1, 108.5, 42.6.

NMR spectra

4-((2,2-dimethylhydrazono)methyl)-2-phenylisoindoline-1,3-dione 3a

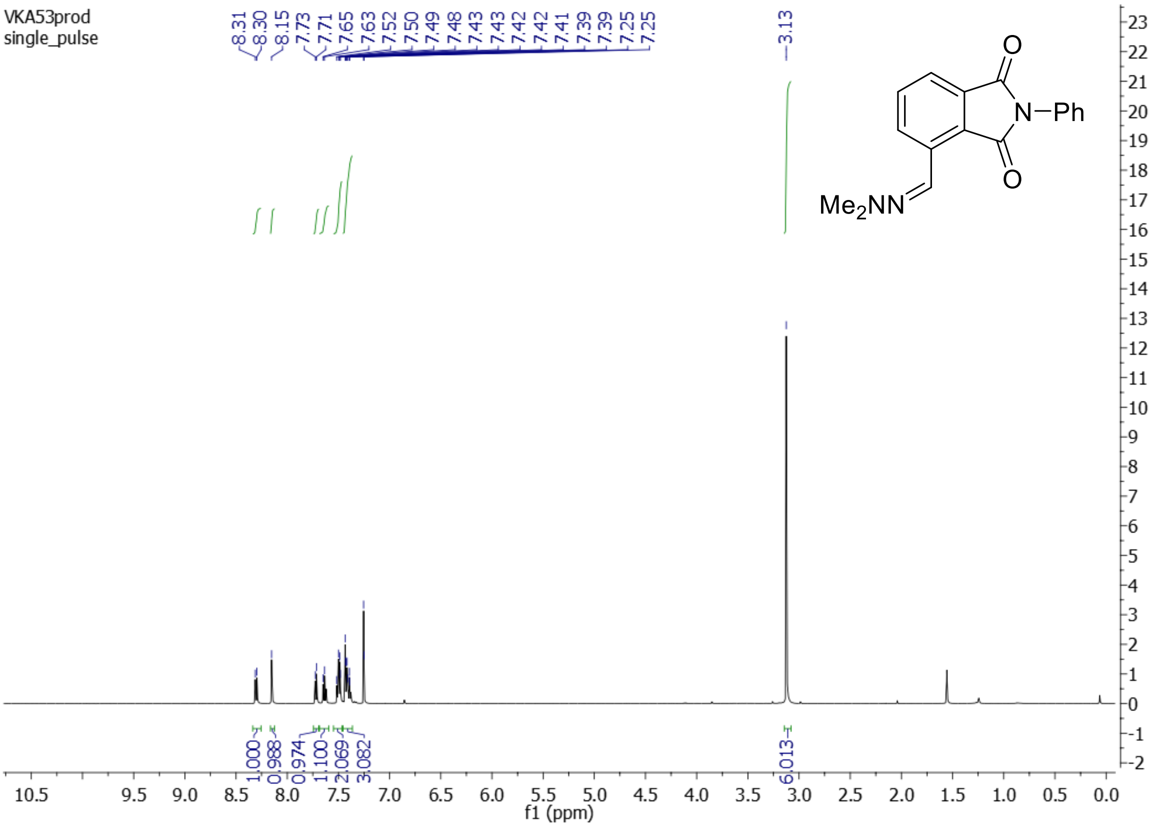

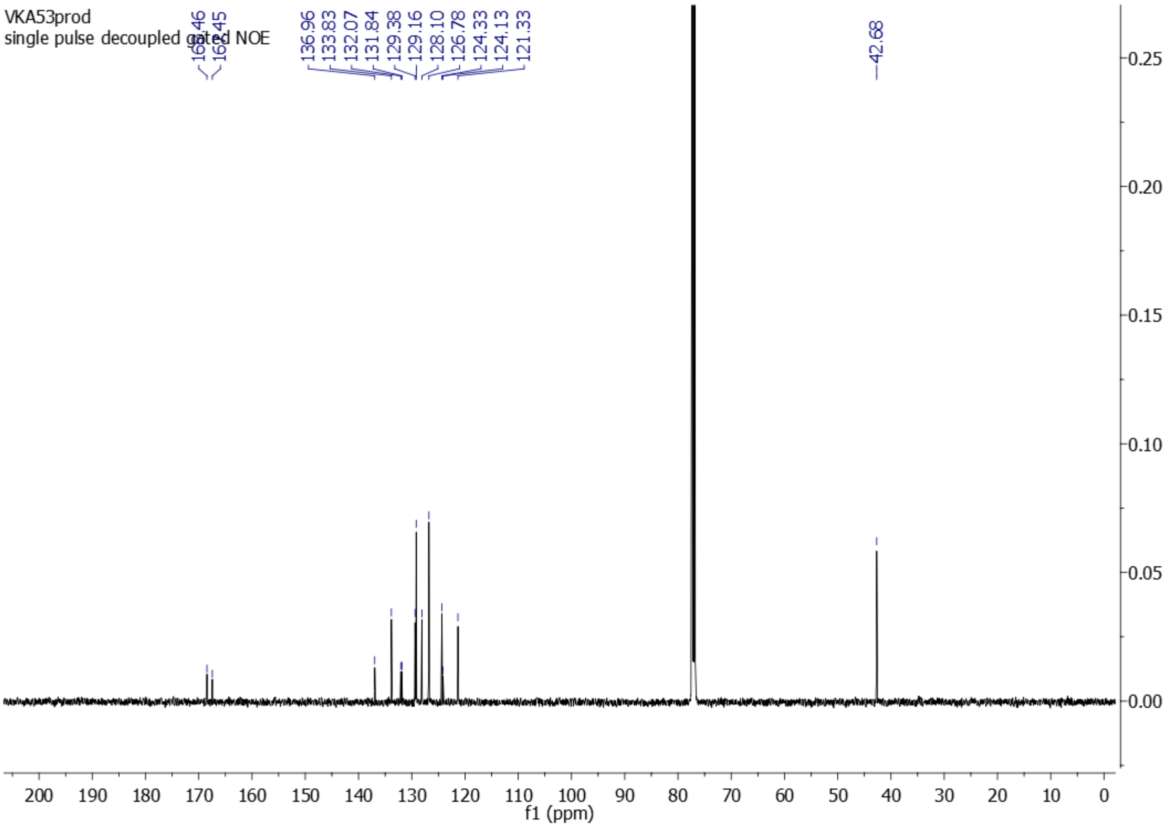

4-((2,2-Dimethylhydrazono)methyl)-2-ethylisoindoline-1,3-dione 3b

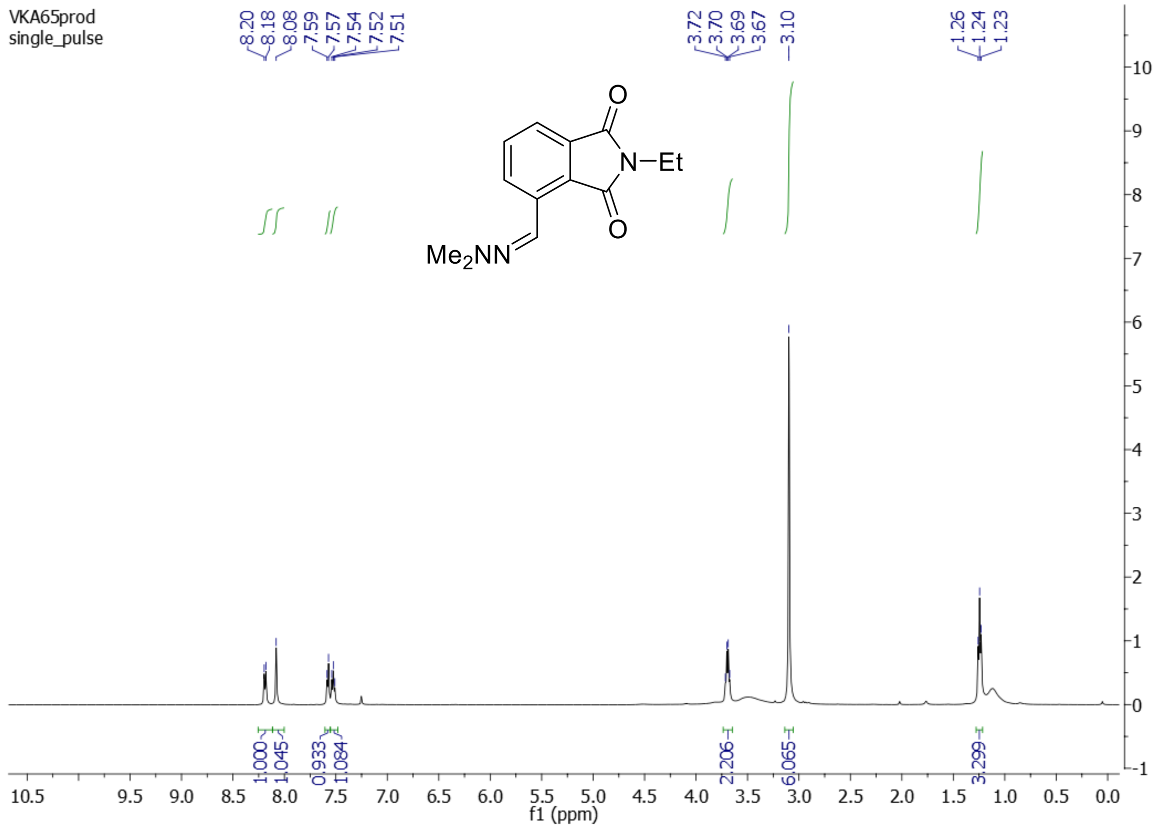

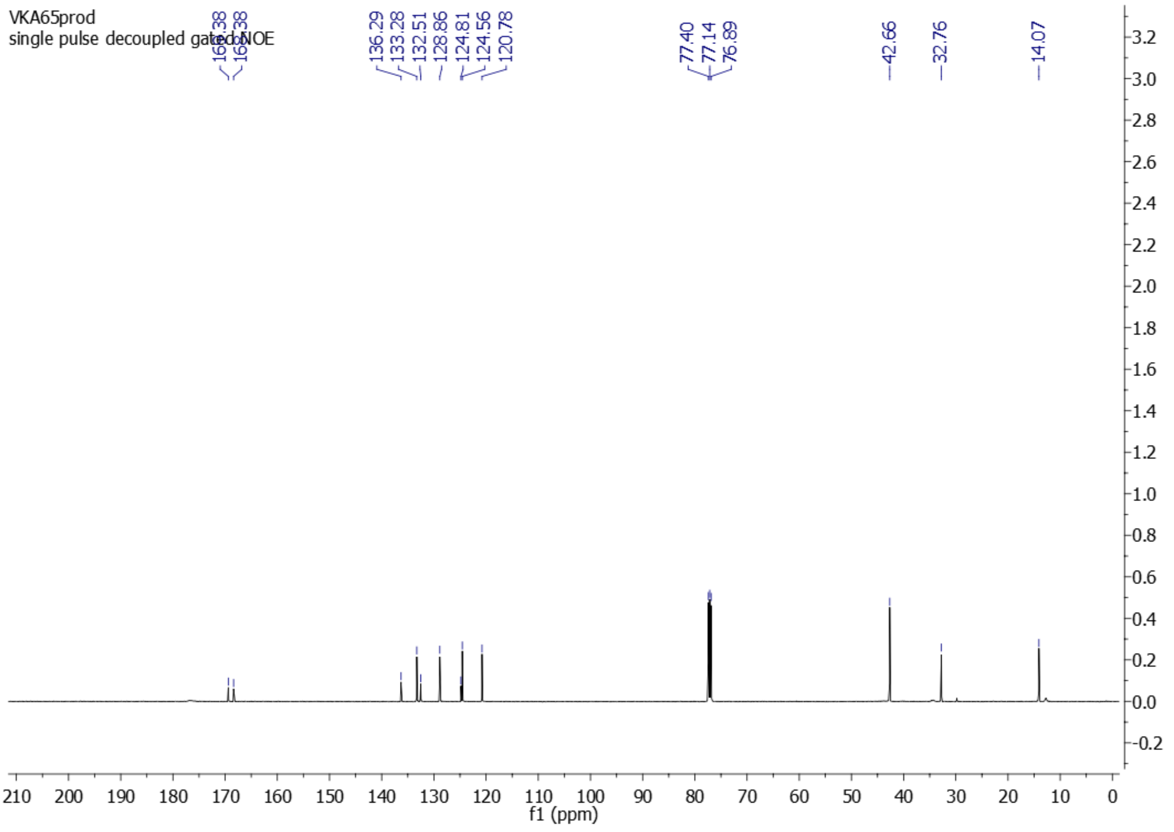

4-((2,2-Dimethylhydrazono)methyl)-2-propylisoindoline-1,3-dione 3c

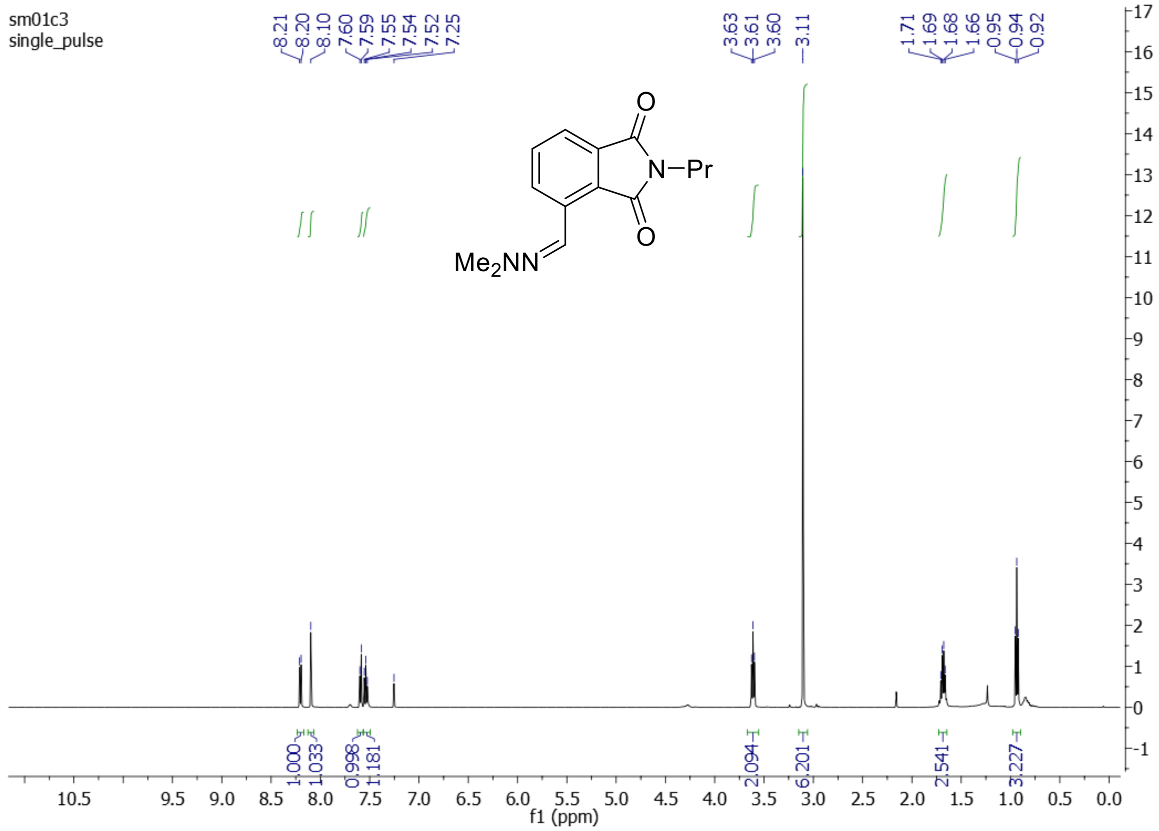

sm01c3 — single pulse decoupled gated NOE

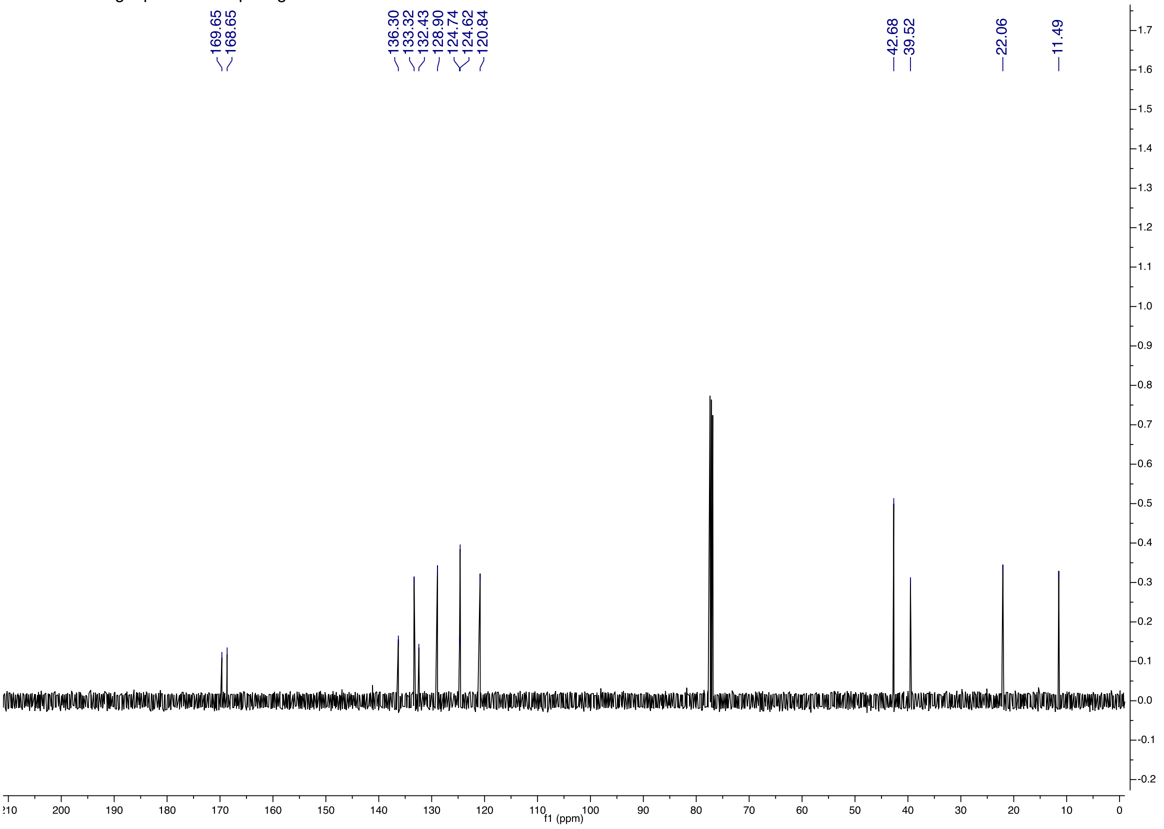

2-Butyl-4-((2,2-dimethylhydrazono)methyl)isoindoline-1,3-dione 3d

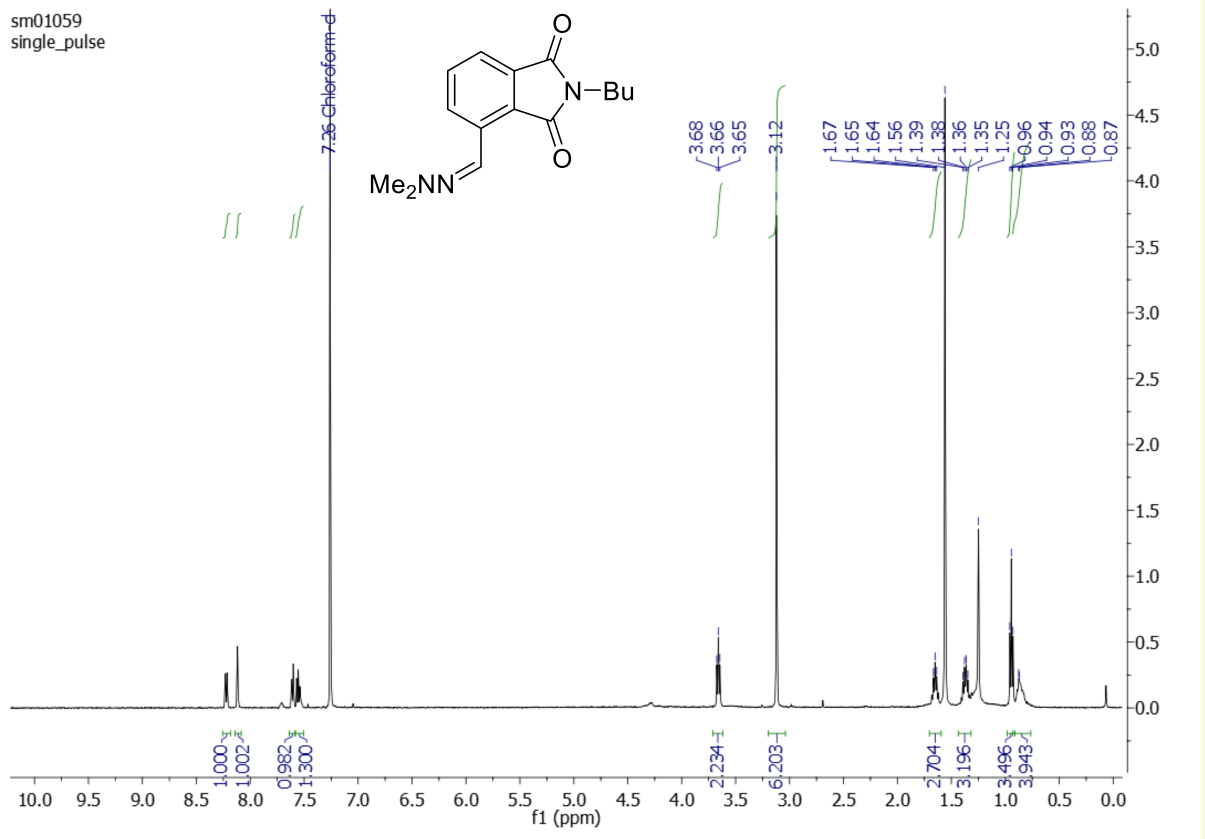

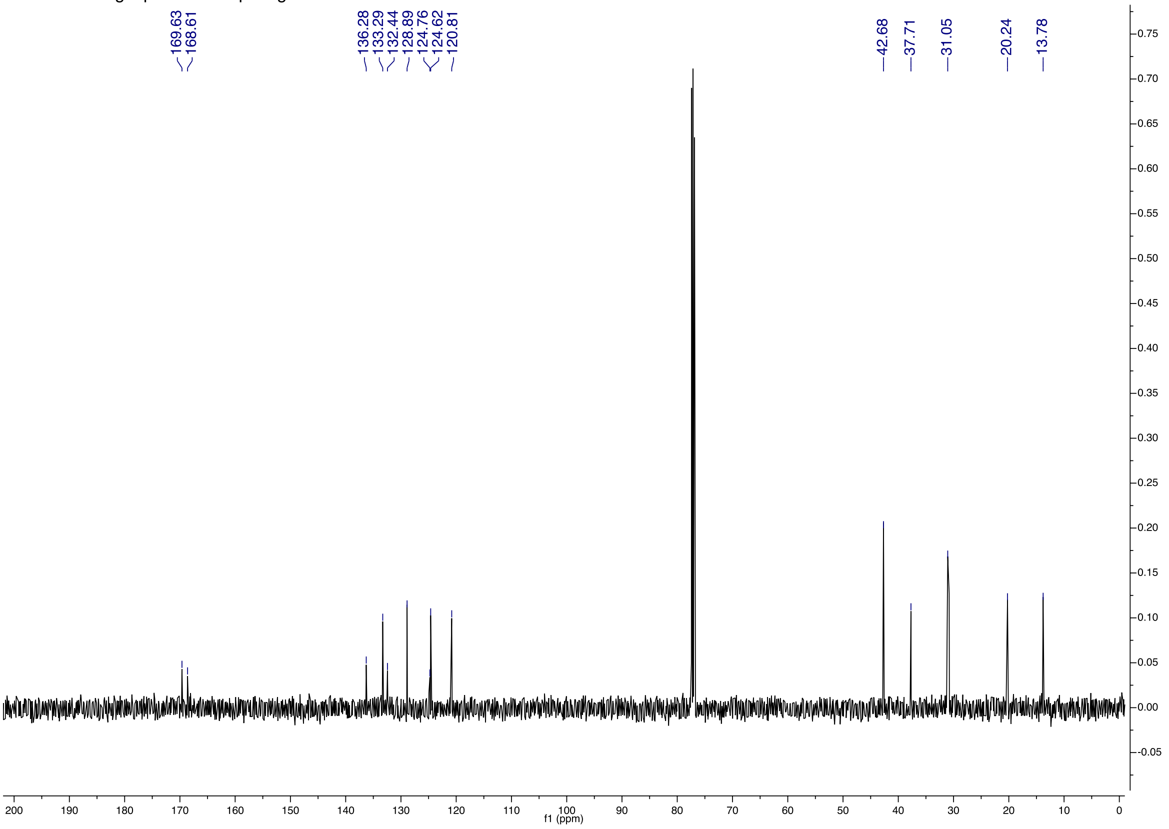

4-((2,2-Dimethylhydrazono)methyl)-2-pentylisoindoline-1,3-dione 3e

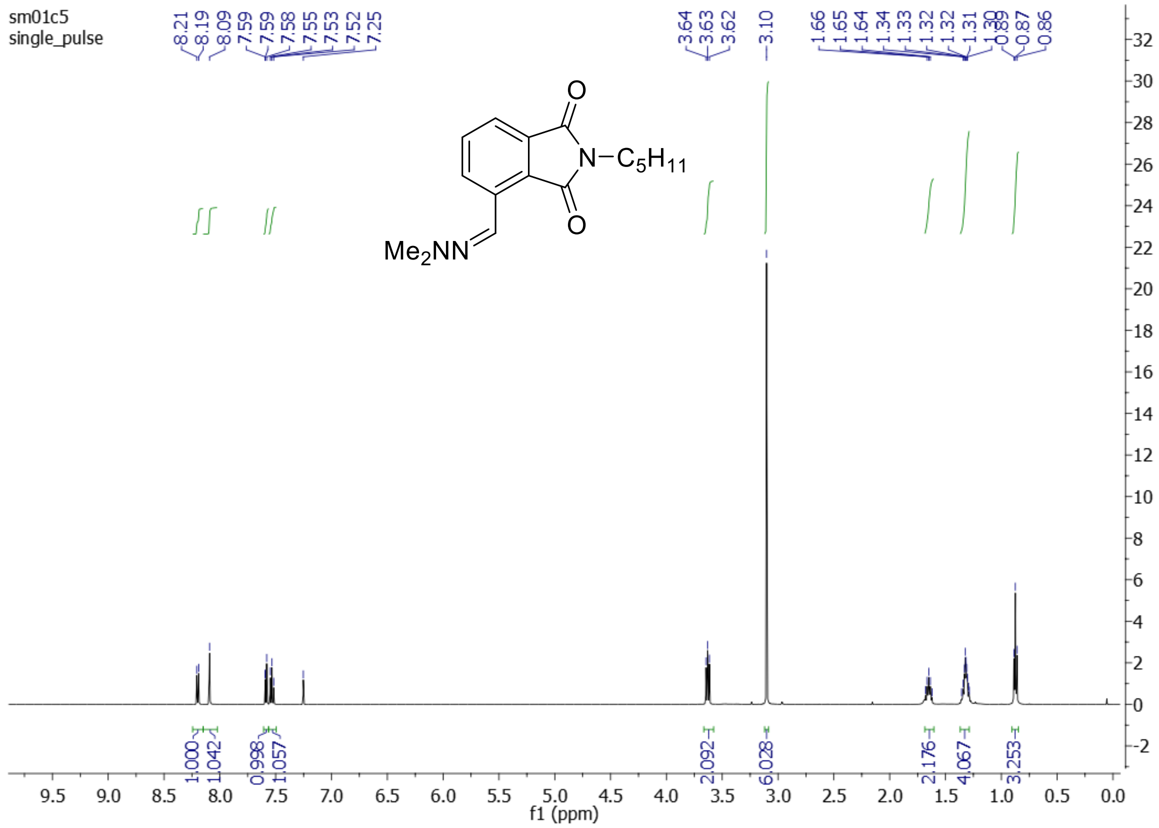

sm01c5 — single pulse decoupled gated NOE

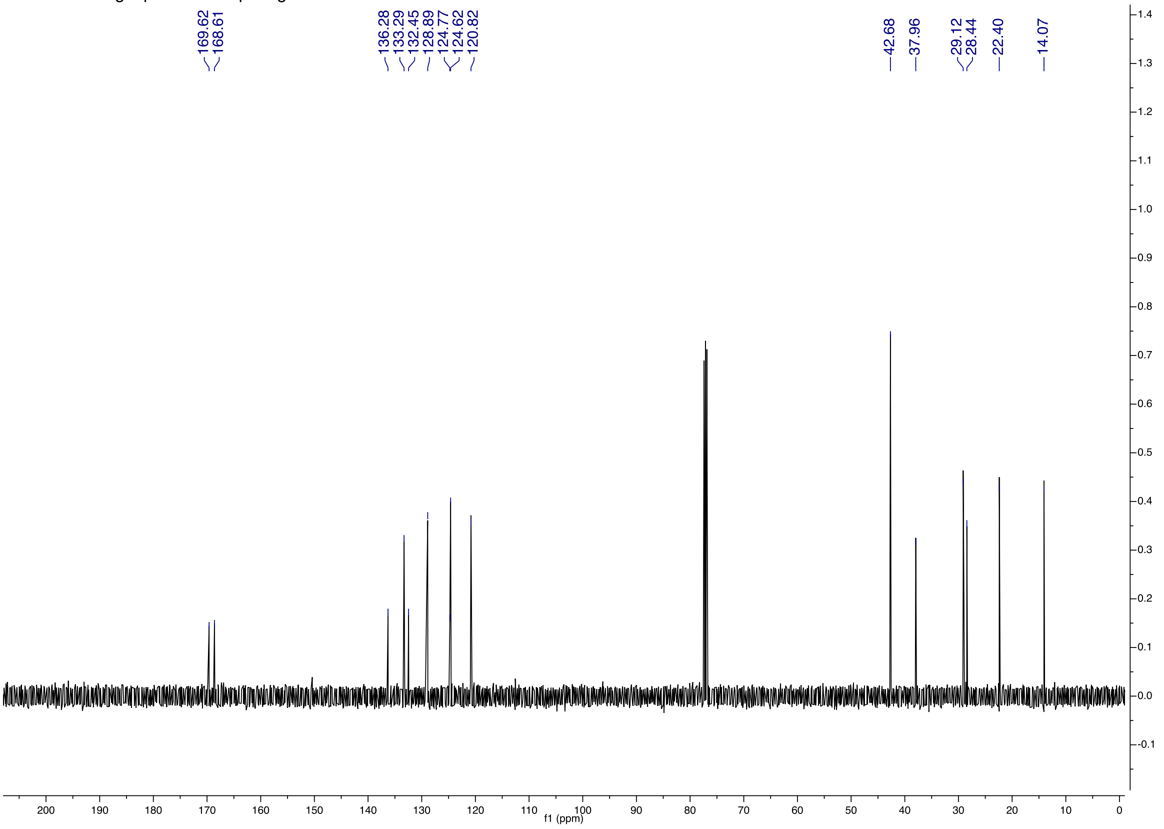

4-((2,2-Dimethylhydrazono)methyl)-2-hexylisoindoline-1,3-dione 3f

yus02027 2C — single_pulse

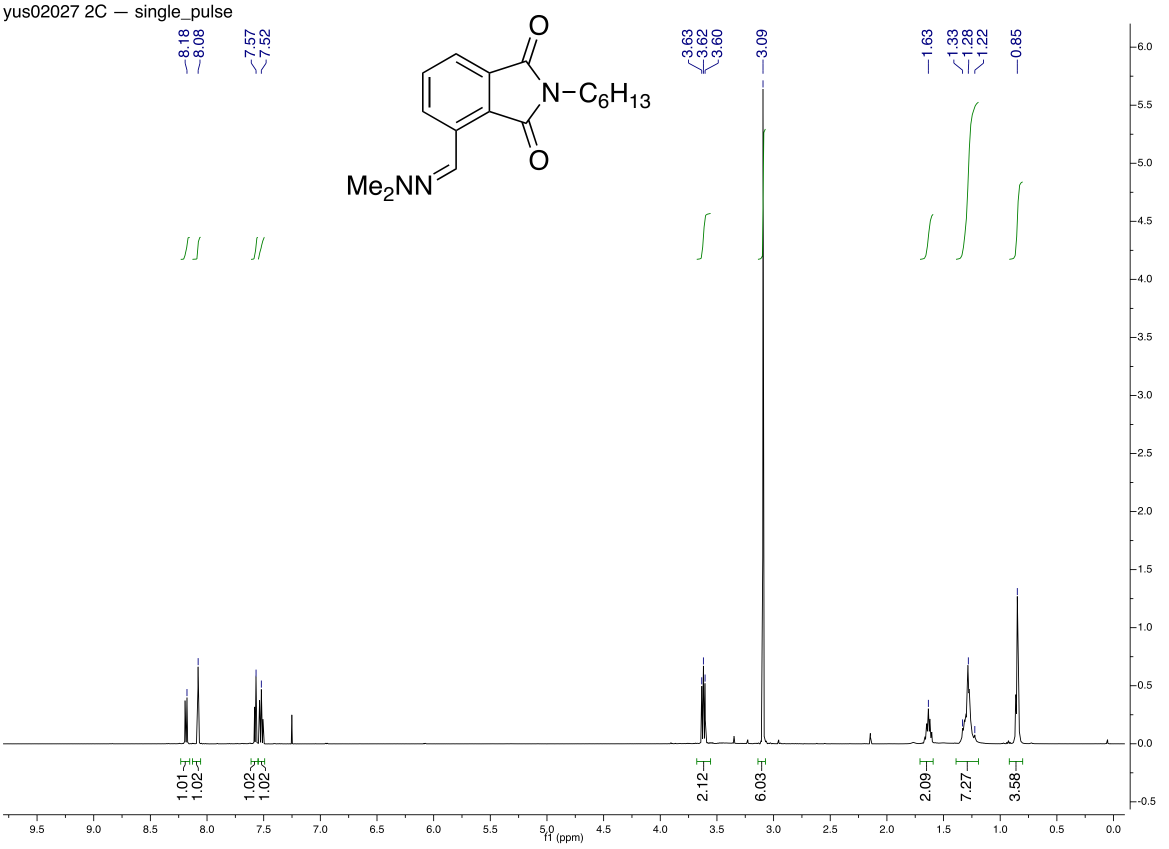

yus02027 2C — single pulse decoupled gated NOE

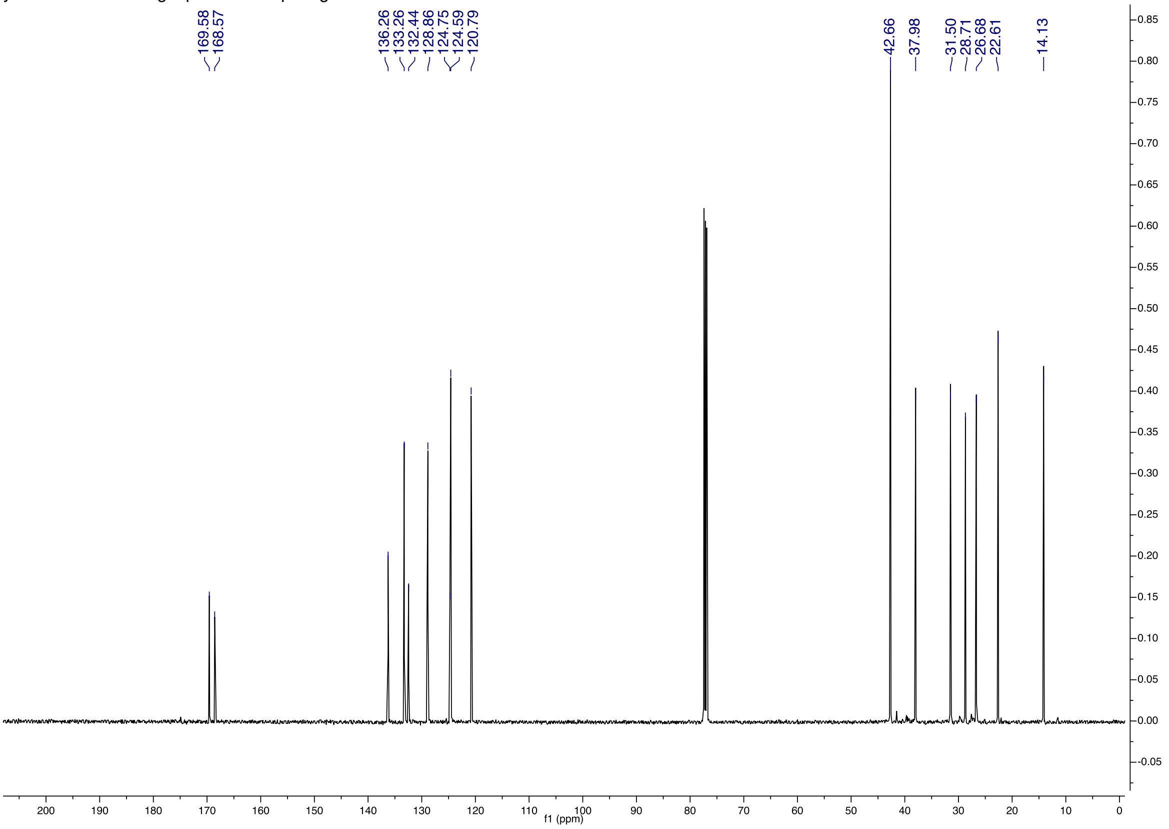

4-((2,2-Dimethylhydrazono)methyl)-2-heptylisoindoline-1,3-dione 3g

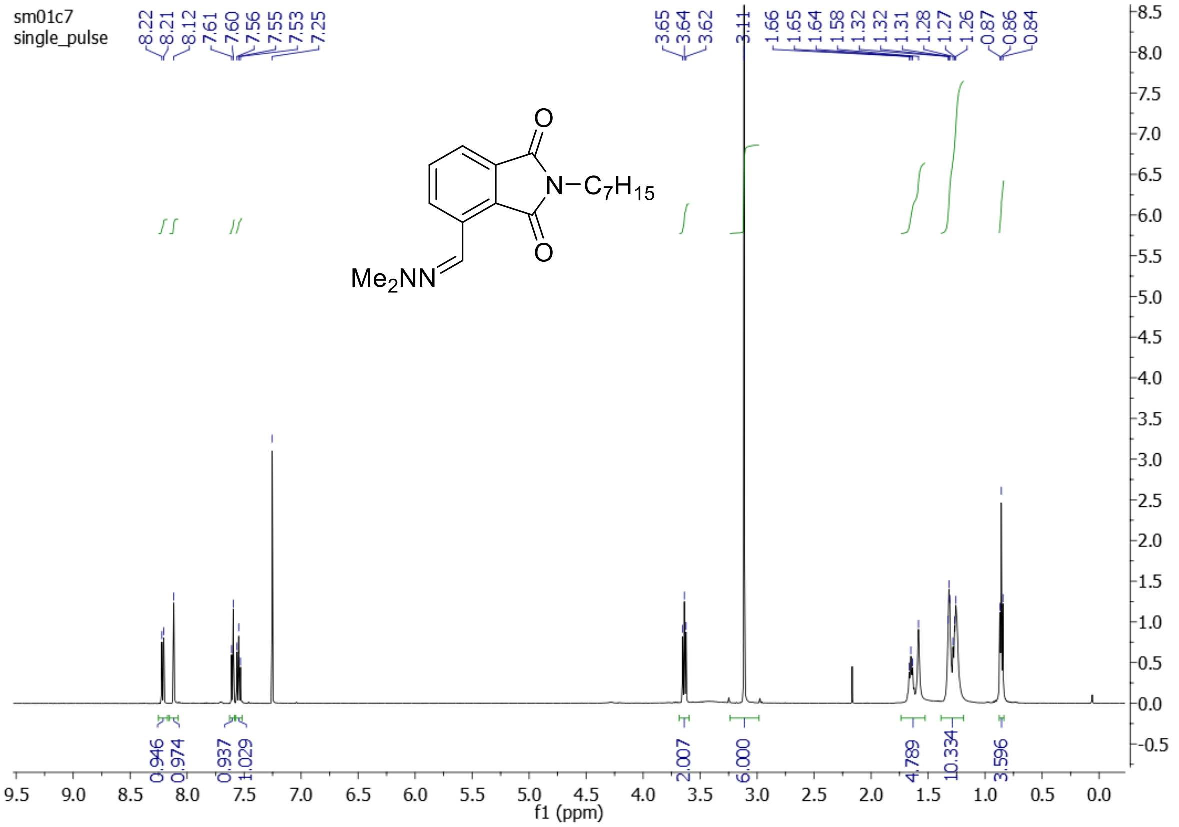

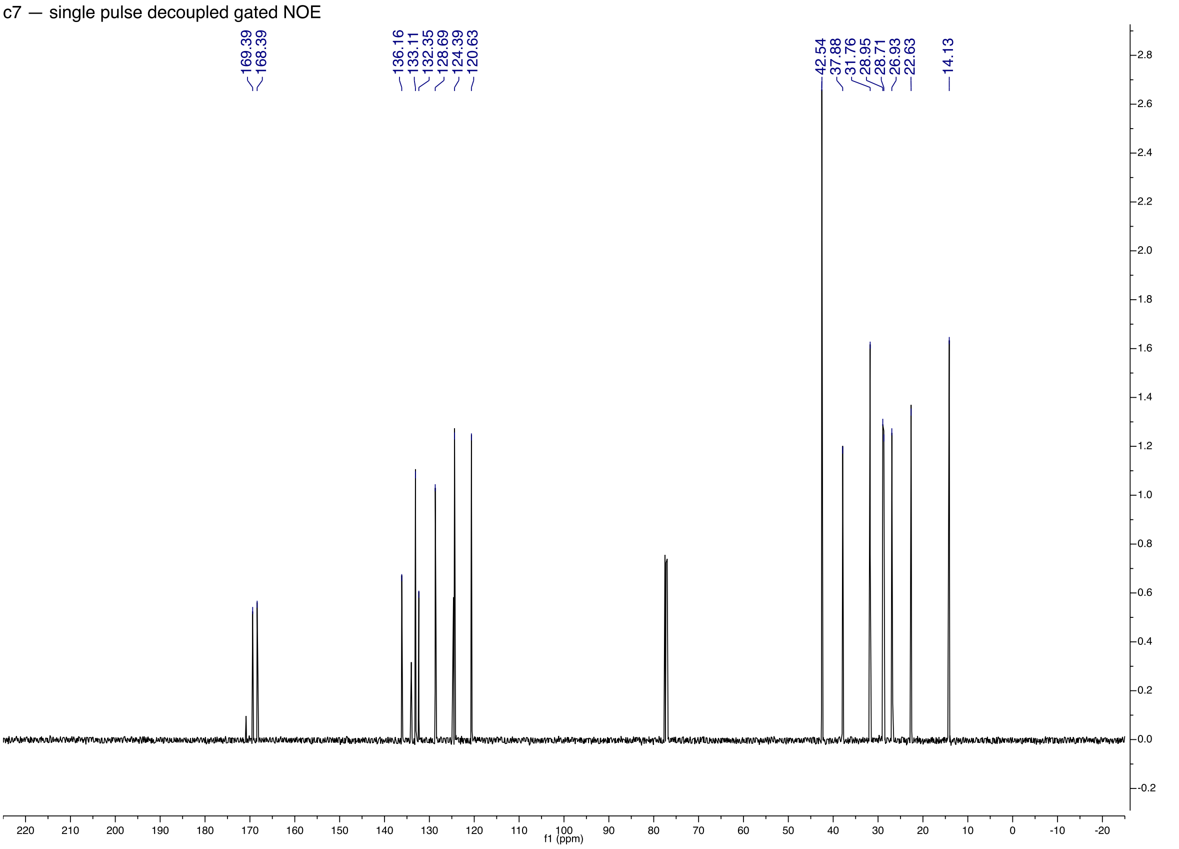

4-((2,2-Dimethylhydrazonoe)methyl)-2-octylisoindoline-1,3-dione 3h

c8h122 — single_pulse

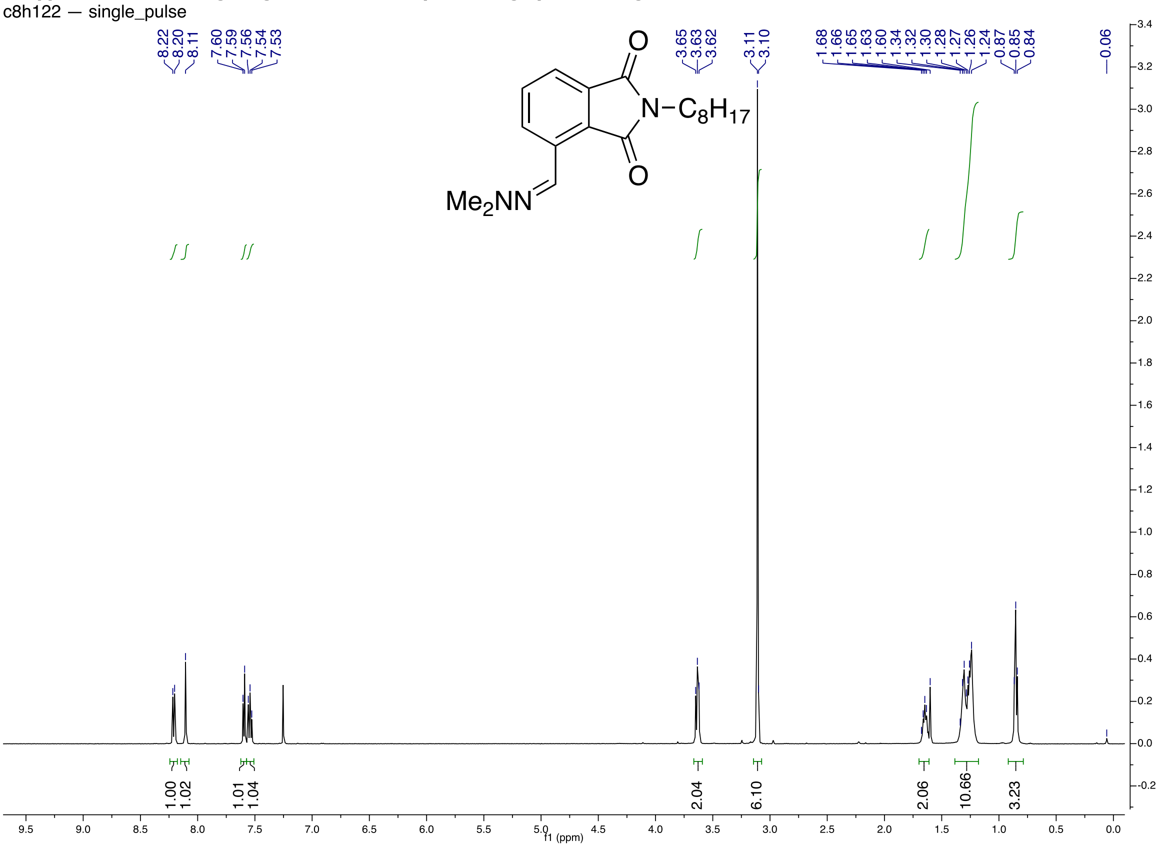

c8c122 2 — single pulse decoupled gated NOE

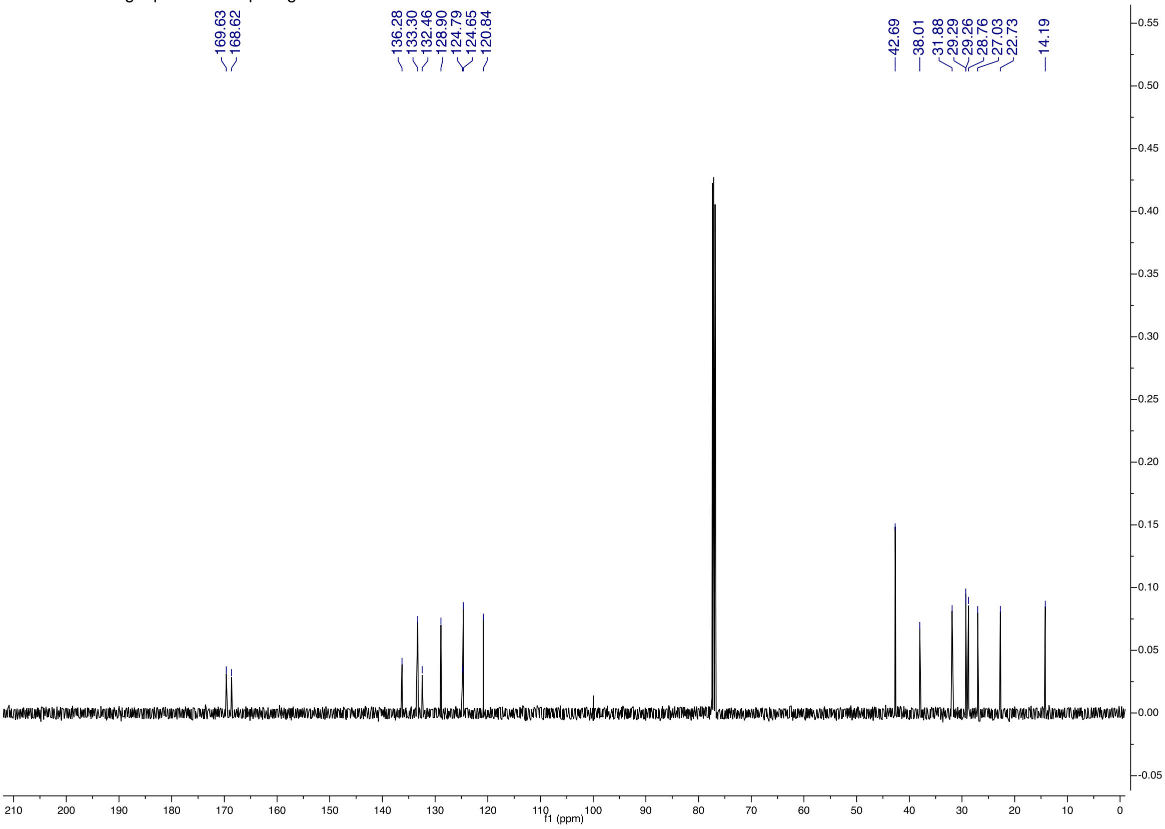

4-((2,2-Dimethylhydrazono)methyl)-2-nonylisoindoline-1,3-dione 3i

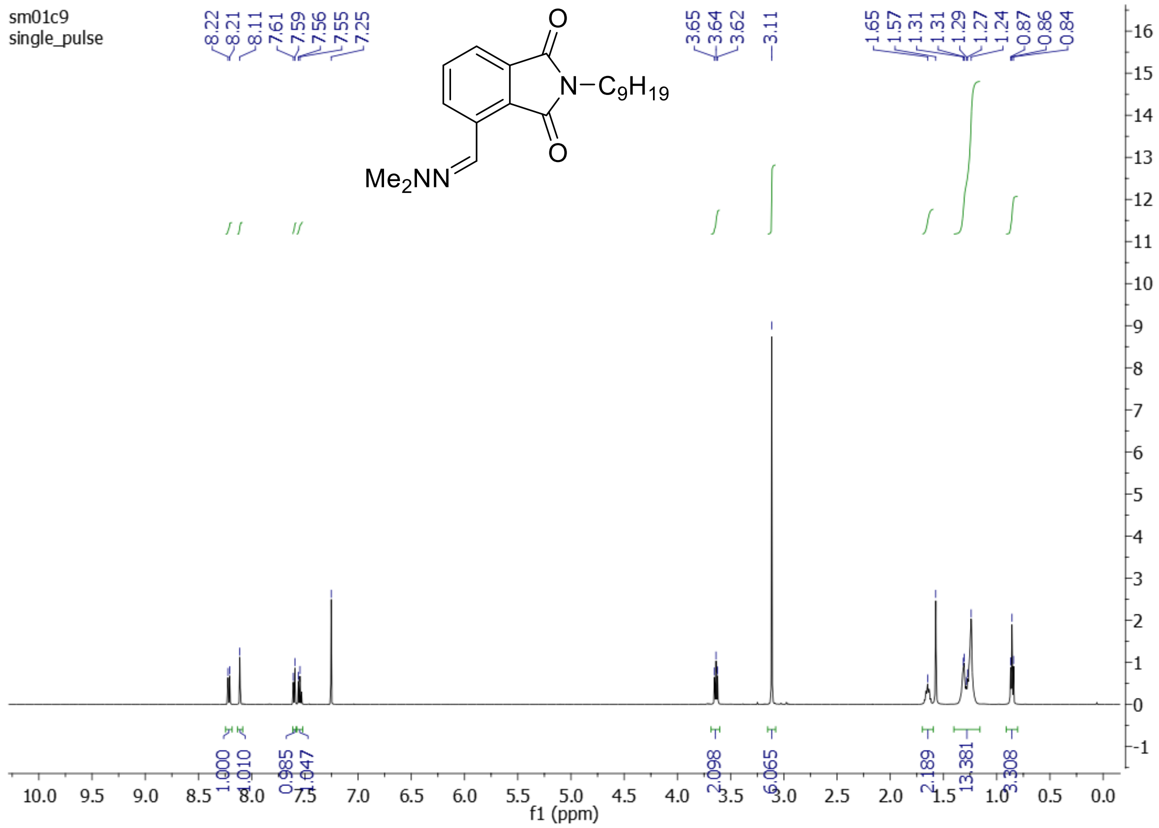

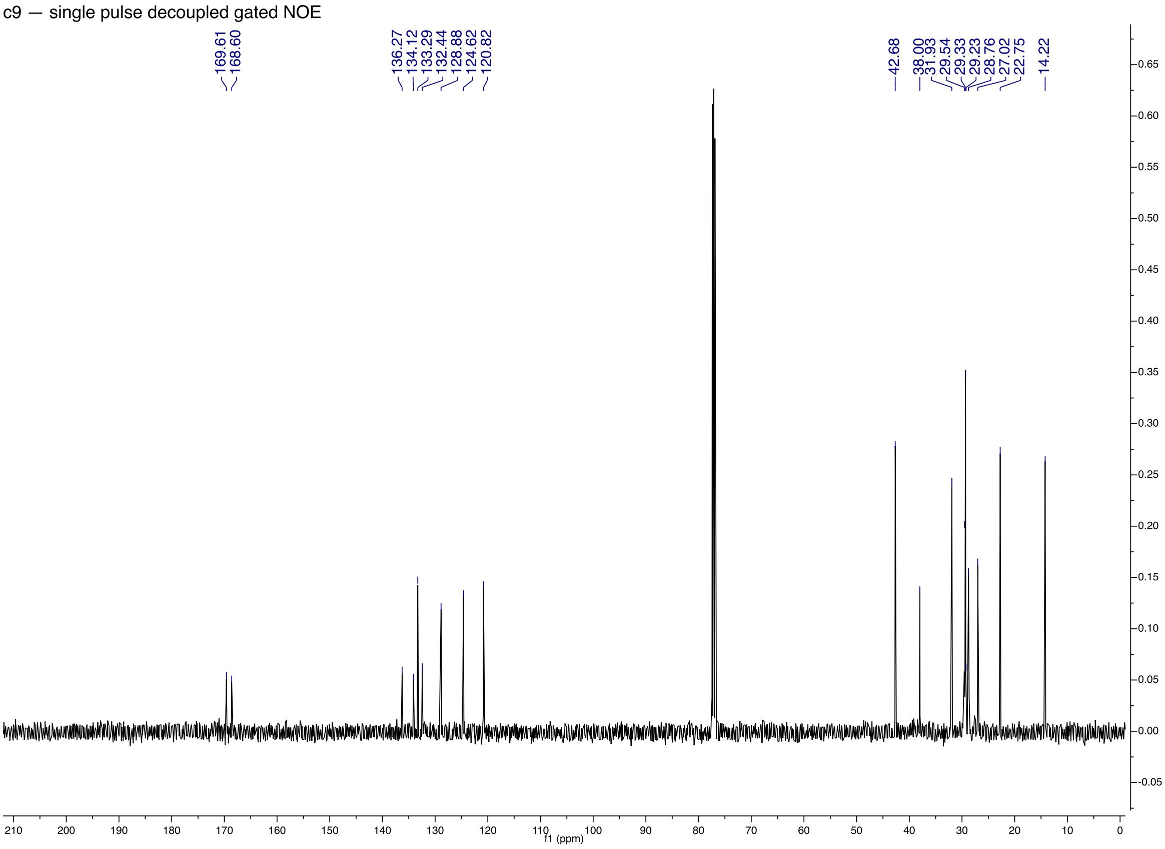

4-((2,2-Dimethylhydrazono)methyl)isoindoline-1,3-dione 3j

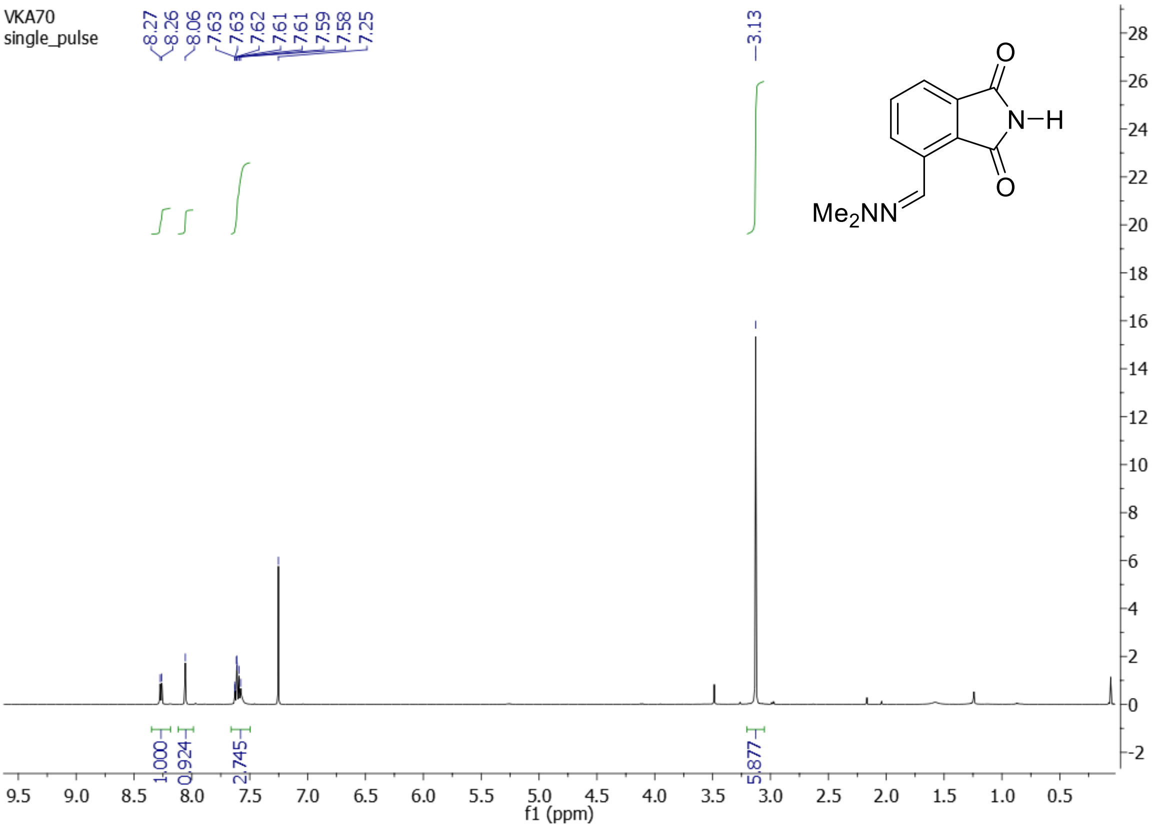

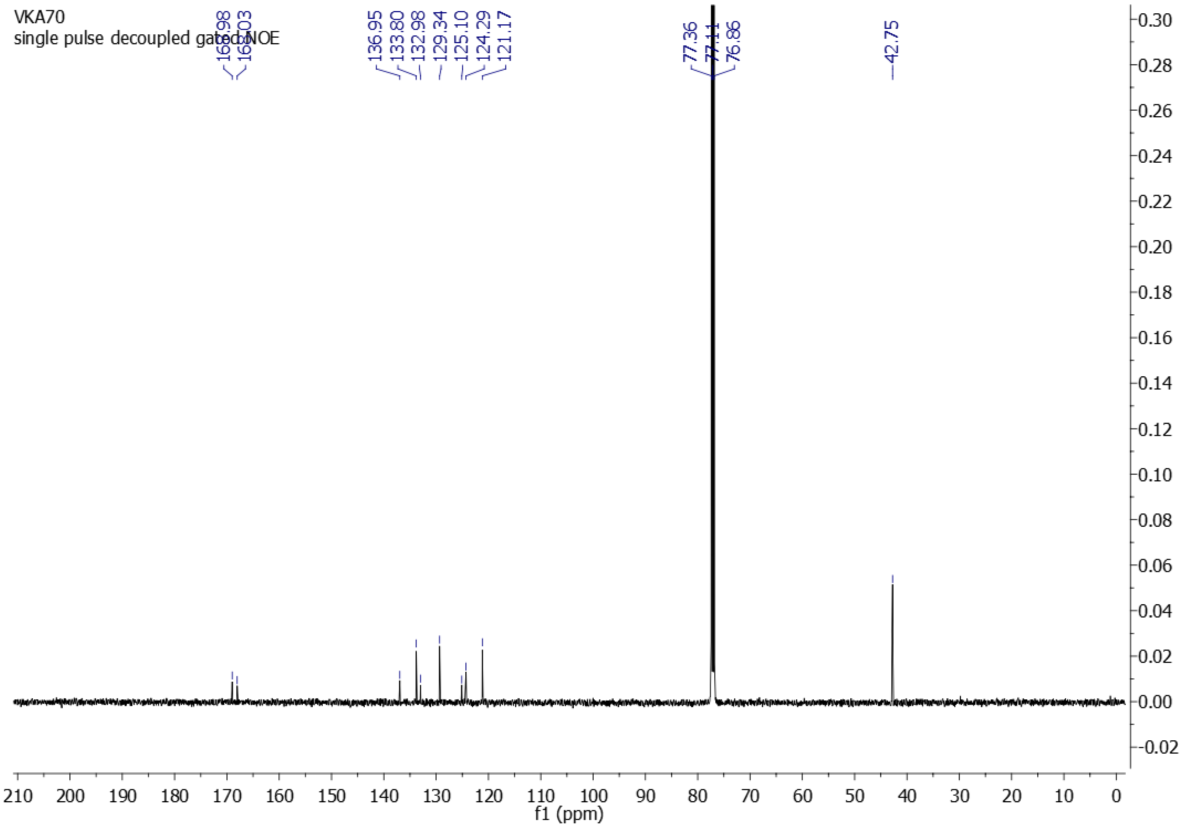

2-Cyclohexyl-4-((2,2-dimethylhydrazono)methyl)isoindoline-1,3-dione 3k

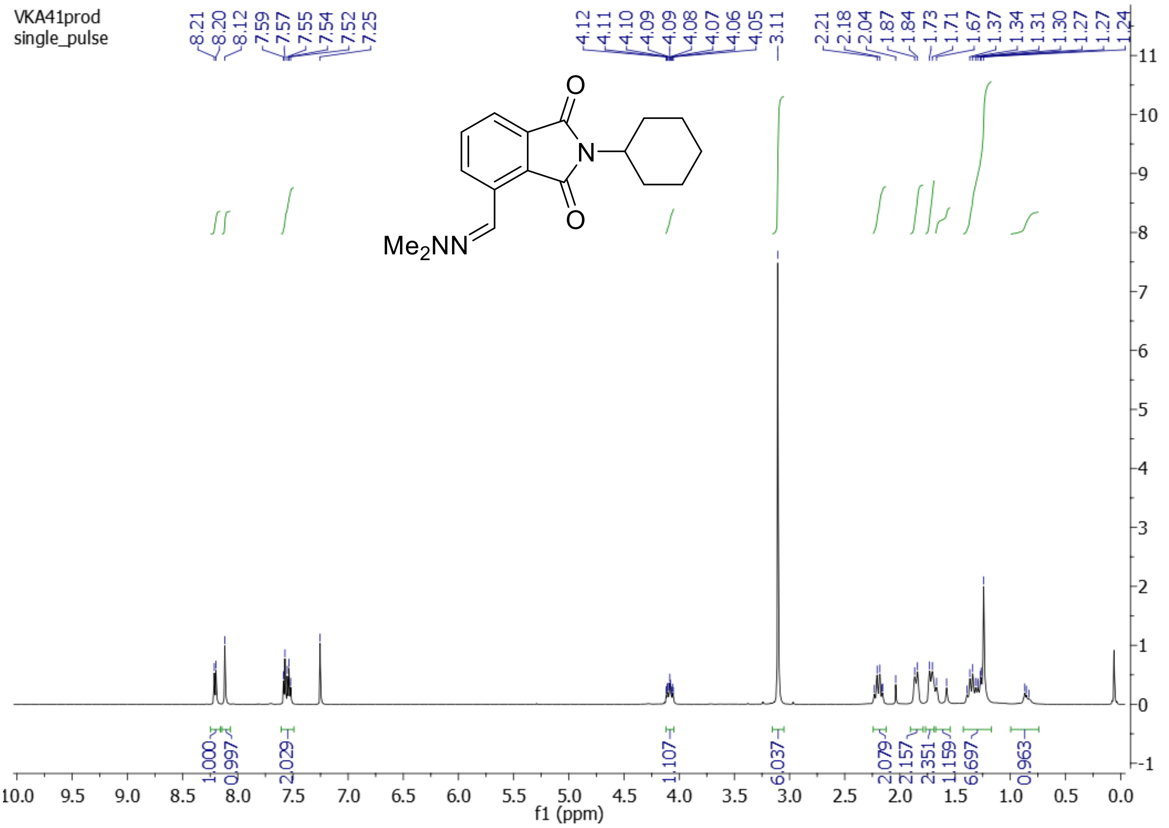

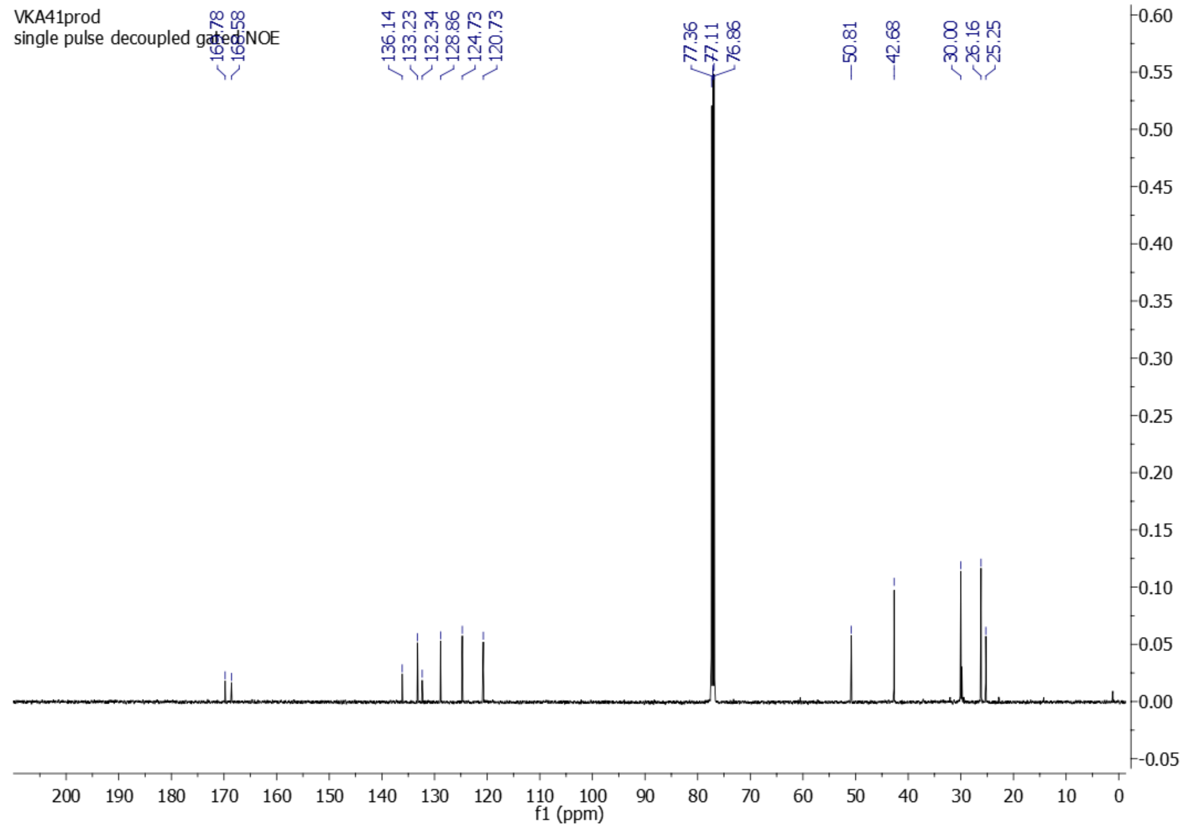

2-Allyl-4-((2,2-dimethylhydrazono)methyl)isoindoline-1,3-dione 3l

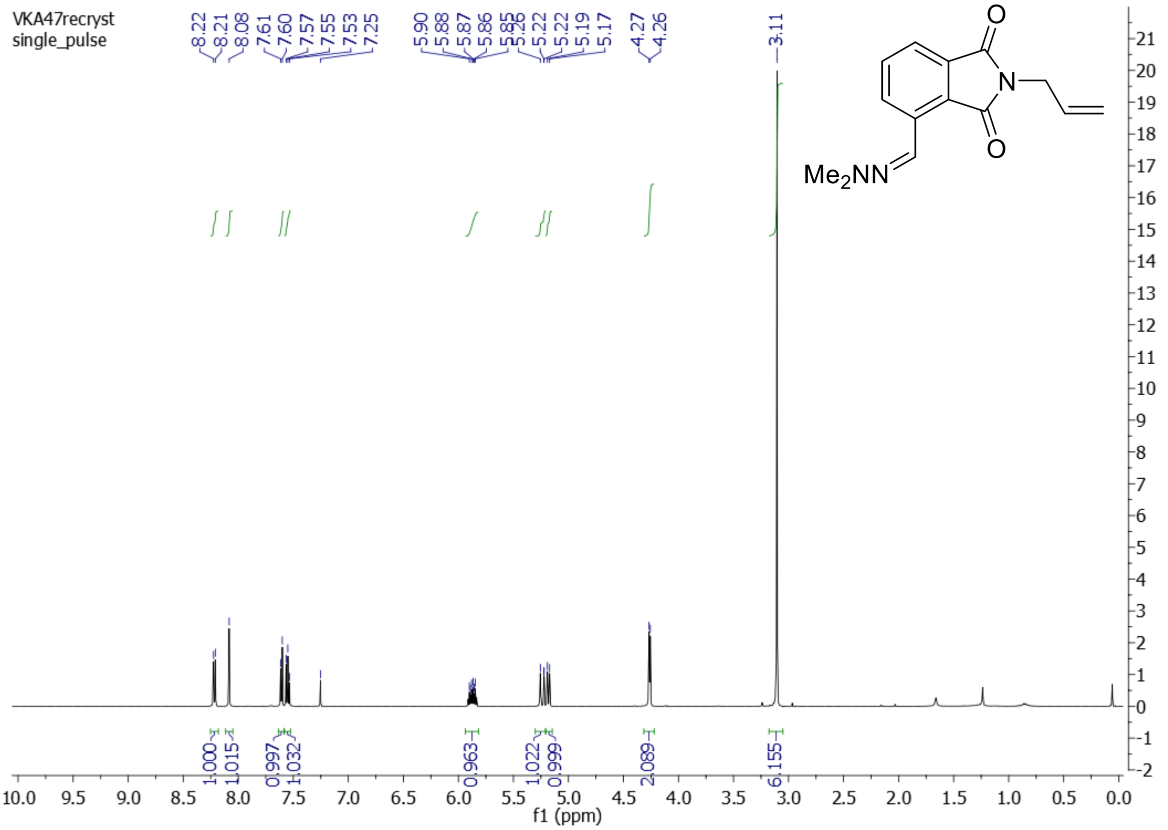

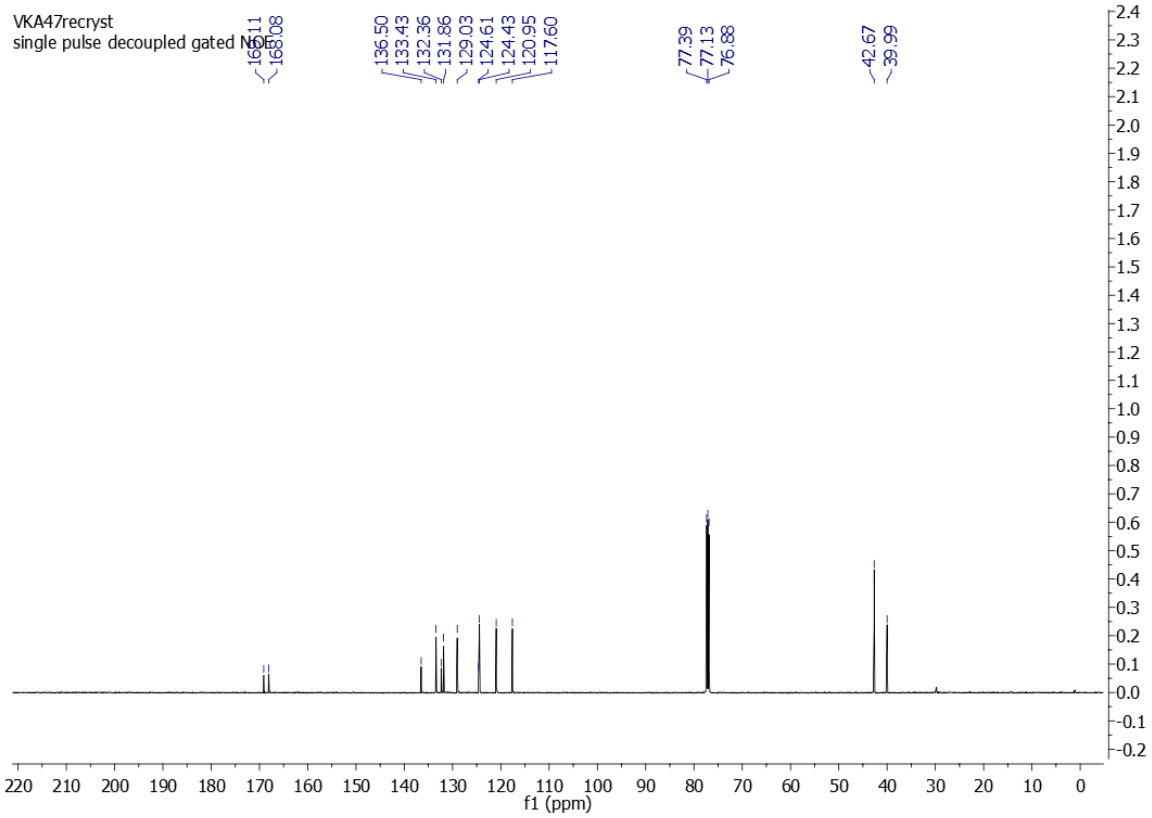

2-Benzyl-4-((2,2-dimethylhydrazono)methyl)isoindoline-1,3-dione 3m

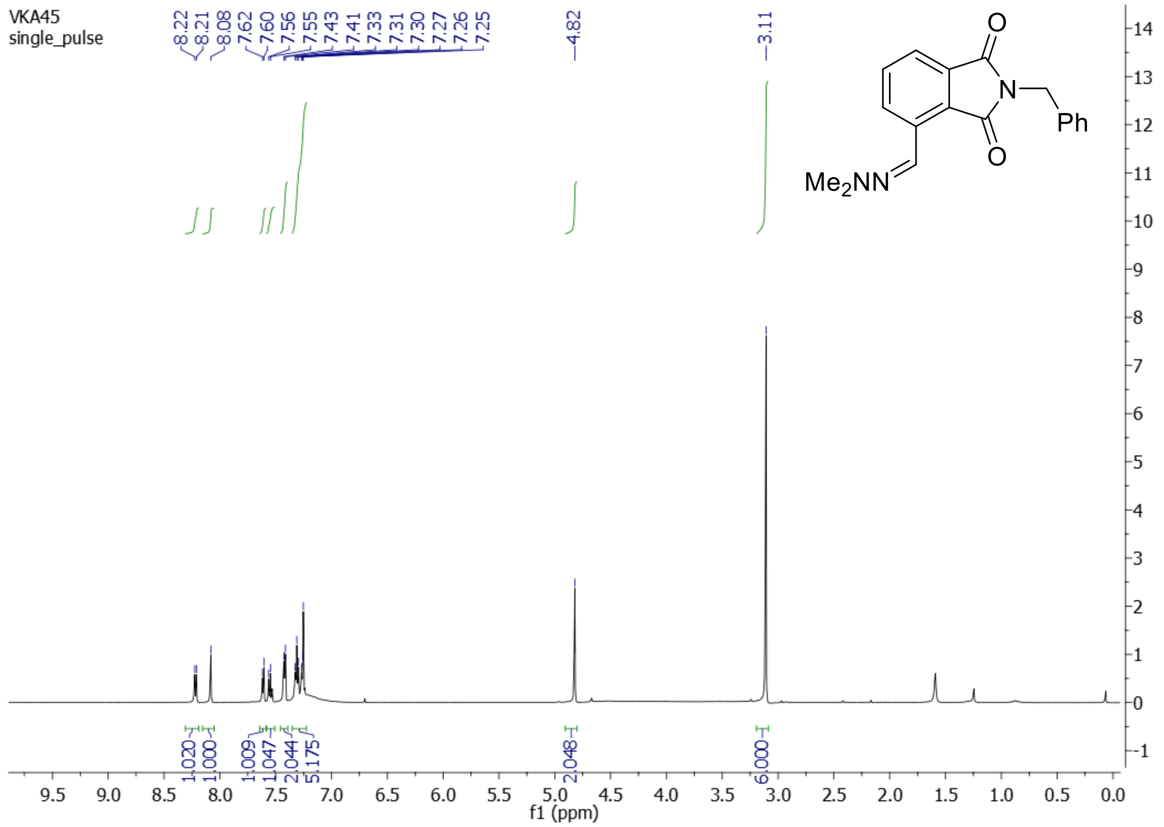

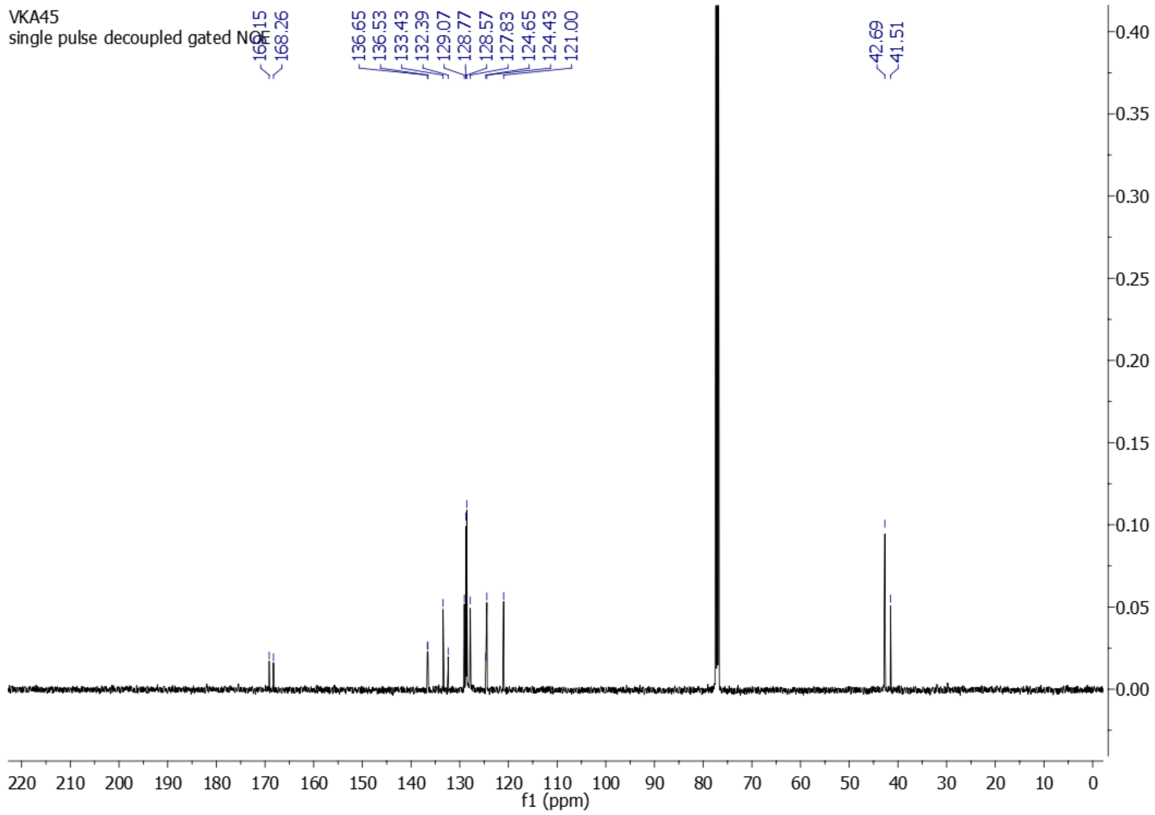

4-((2,2-Dimethylhydrazono)methyl)-2-(4-methoxybenzyl)isoindoline-1,3-dione 3n

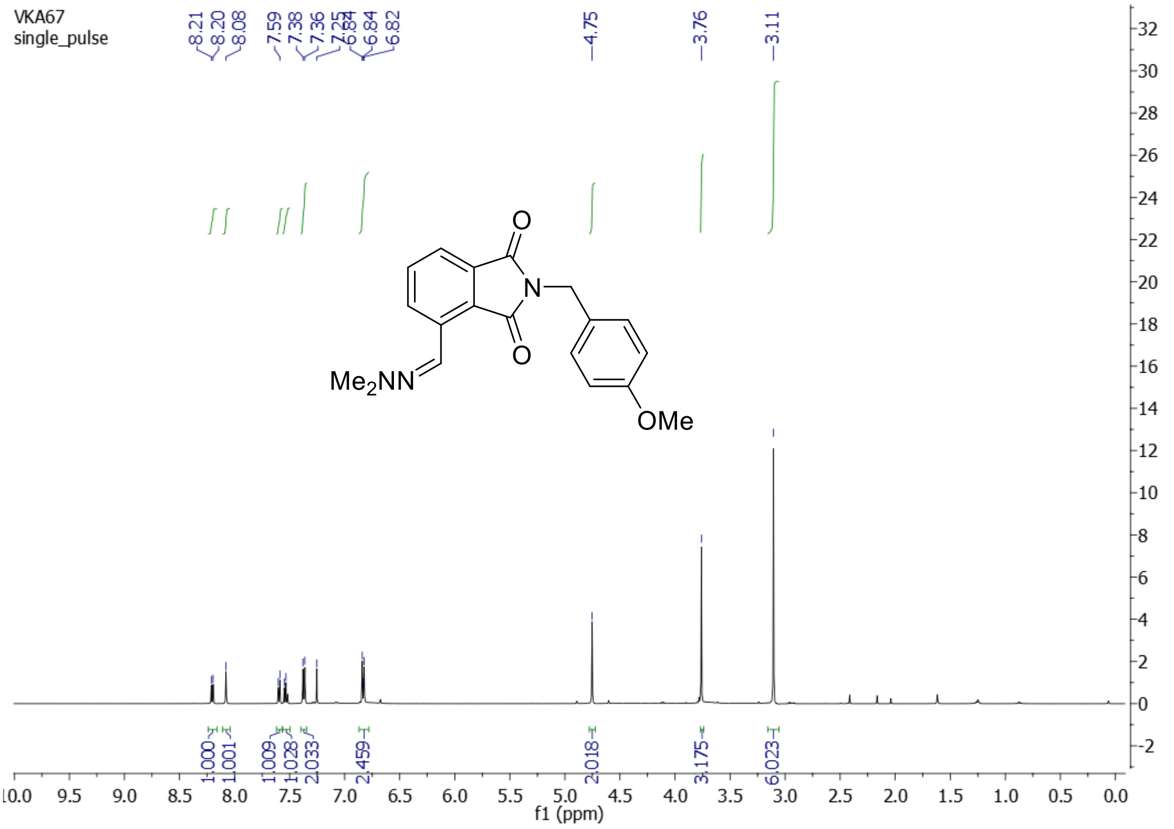

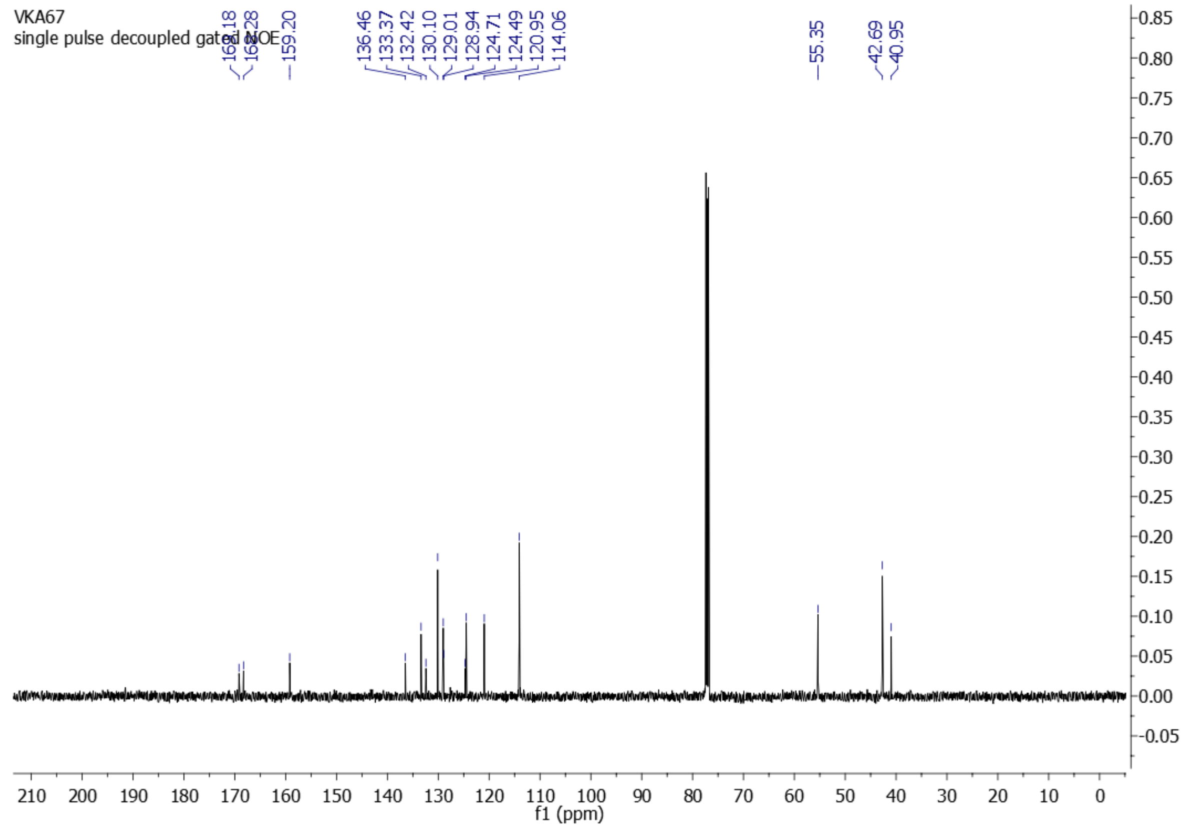

4-((2,2-Dimethylhydrazono)methyl)-2-phenethylisoindoline-1,3-dione 3o

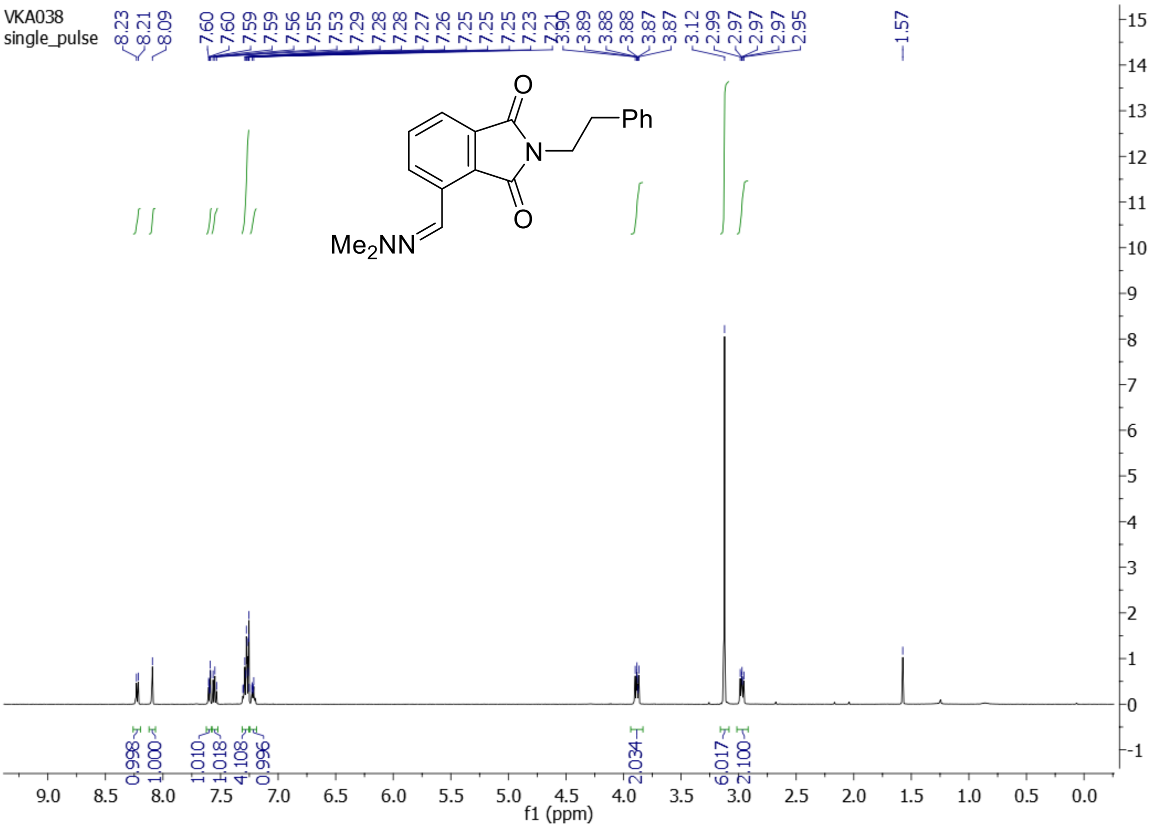

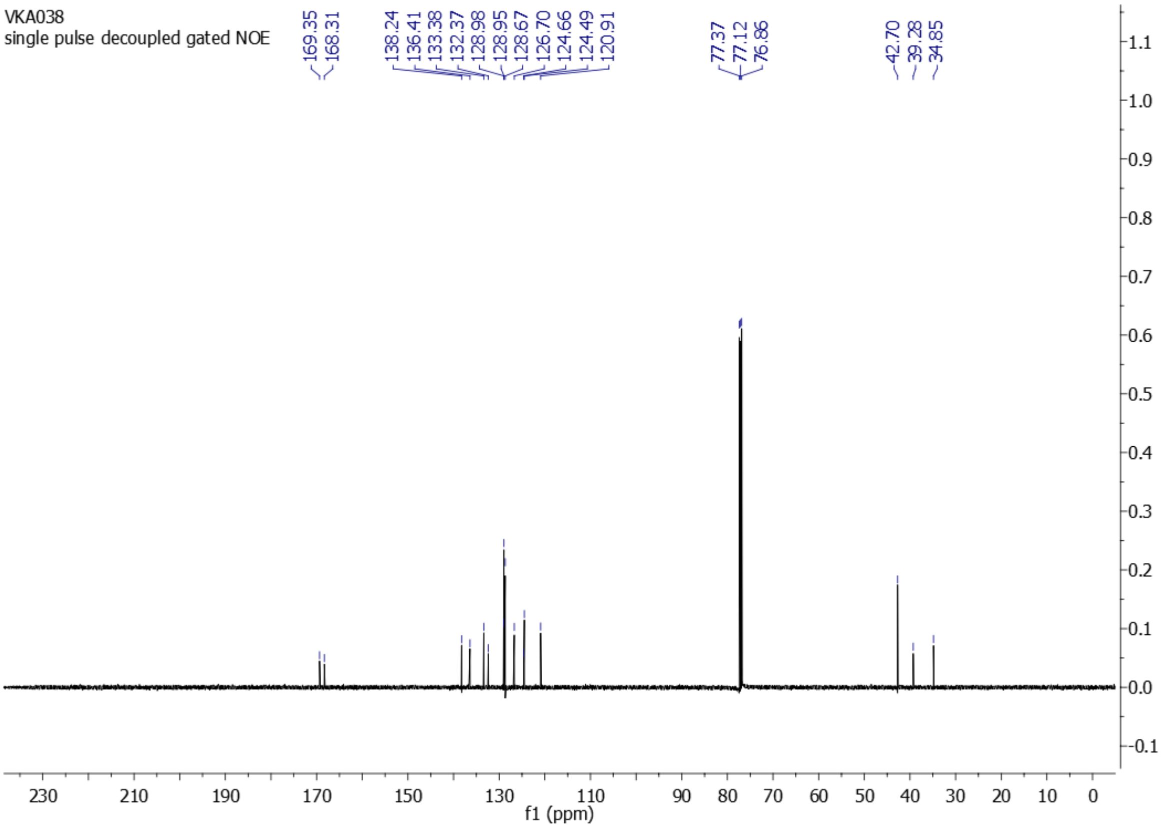

4-((2,2-Dimethylhydrazono)methyl)-2-ethyl-7-methylisoindoline-1,3-dione 3p

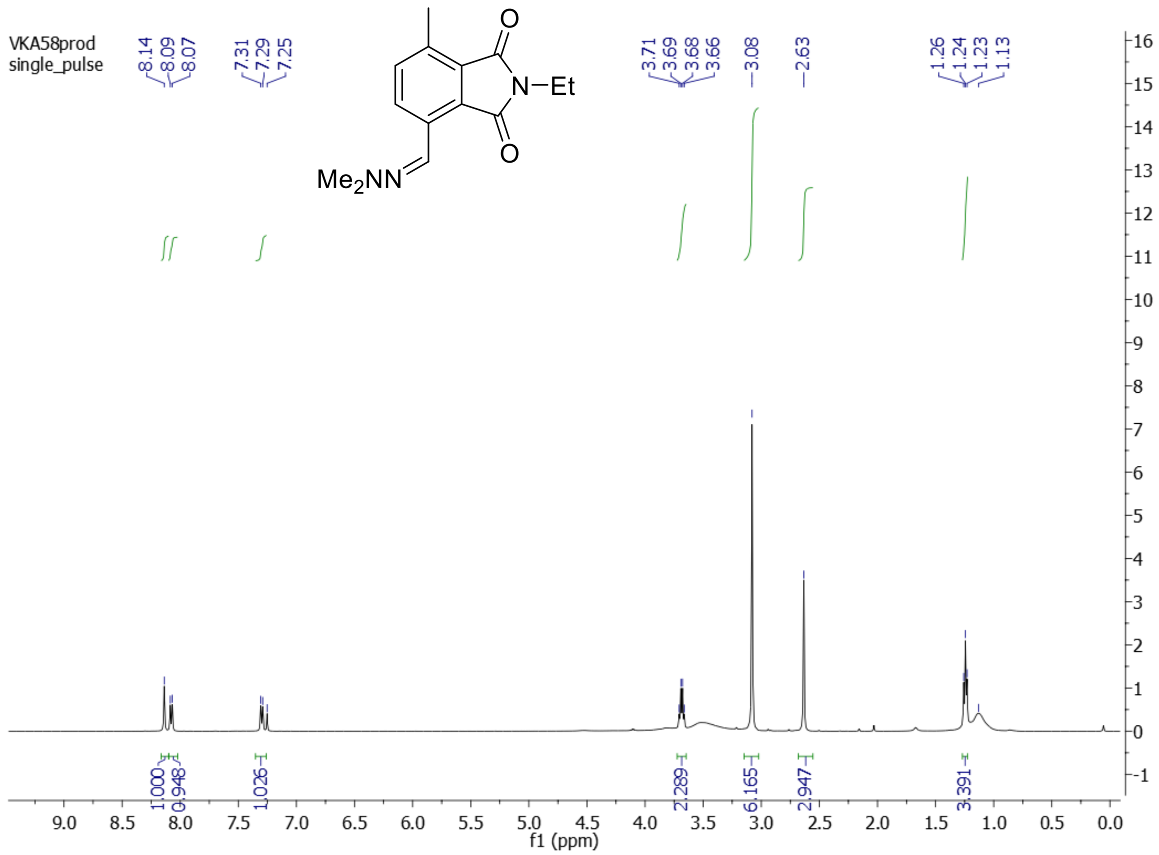

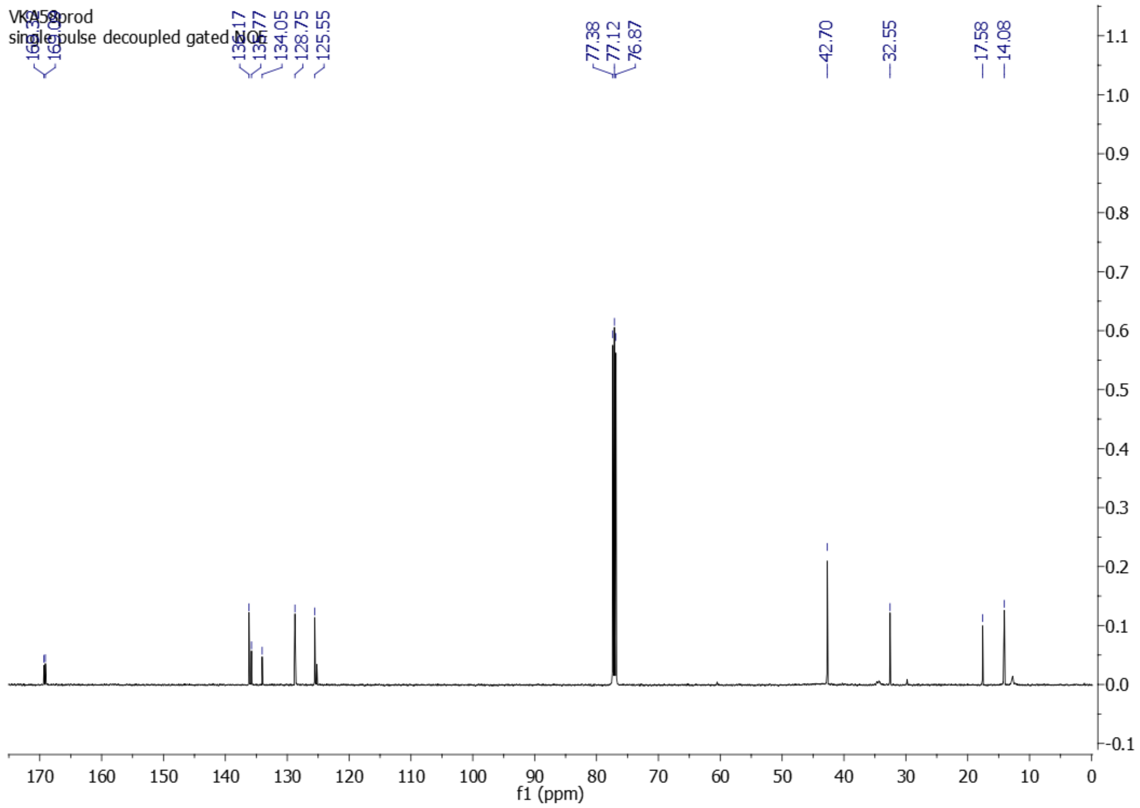

4-((2,2-Dimethylhydrazono)methyl)-7-methyl-2-phenylisoindoline-1,3-dione 3q

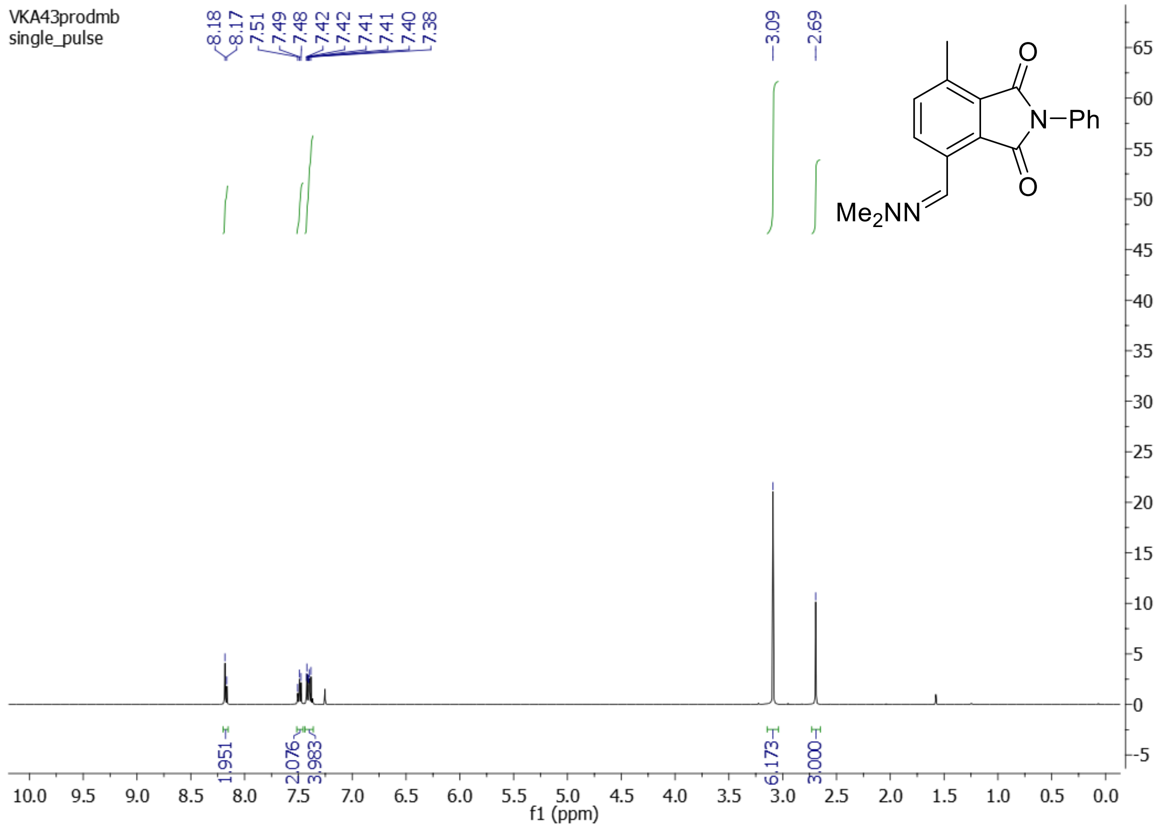

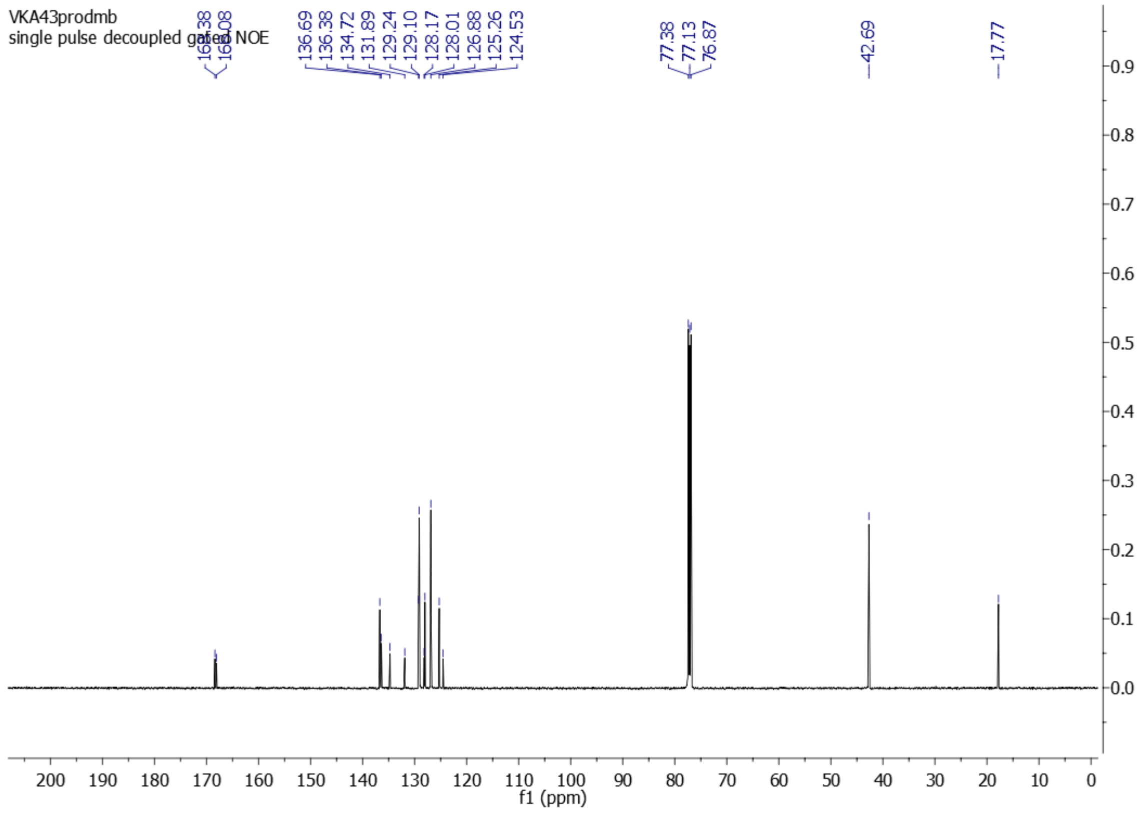

4-((2,2-dimethylhydrazono)methyl)-2-hexyl-7-methylisoindoline-1,3-dione 3r

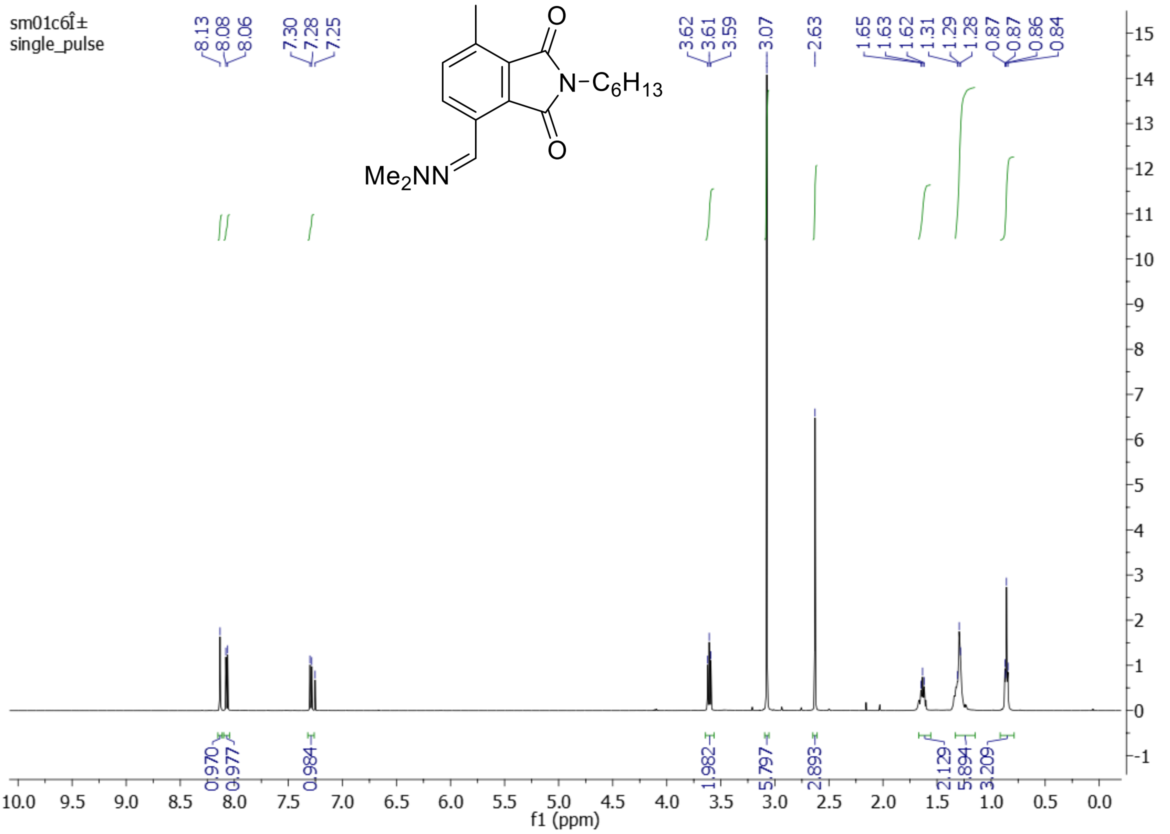

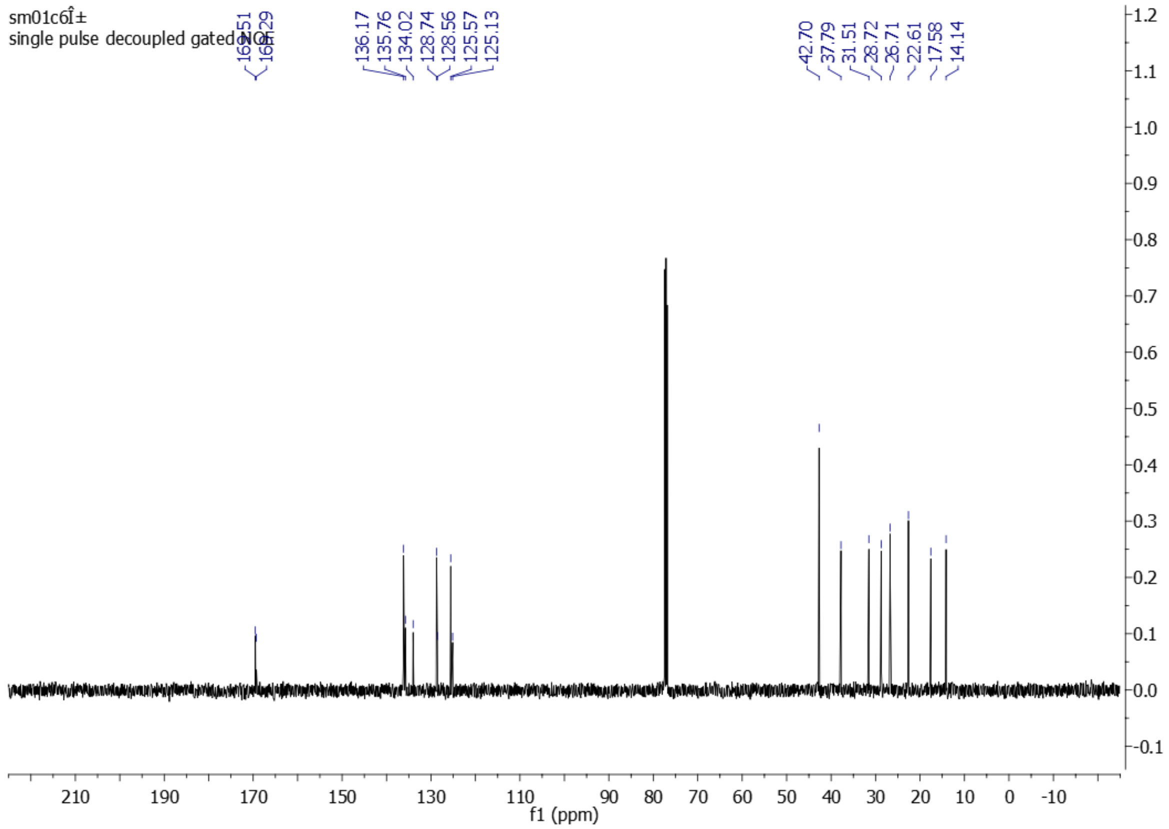

4-((2,2-Dimethylhydrazono)methyl)-2-ethyl-7-hydroxyisoindoline-1,3-dione 3s

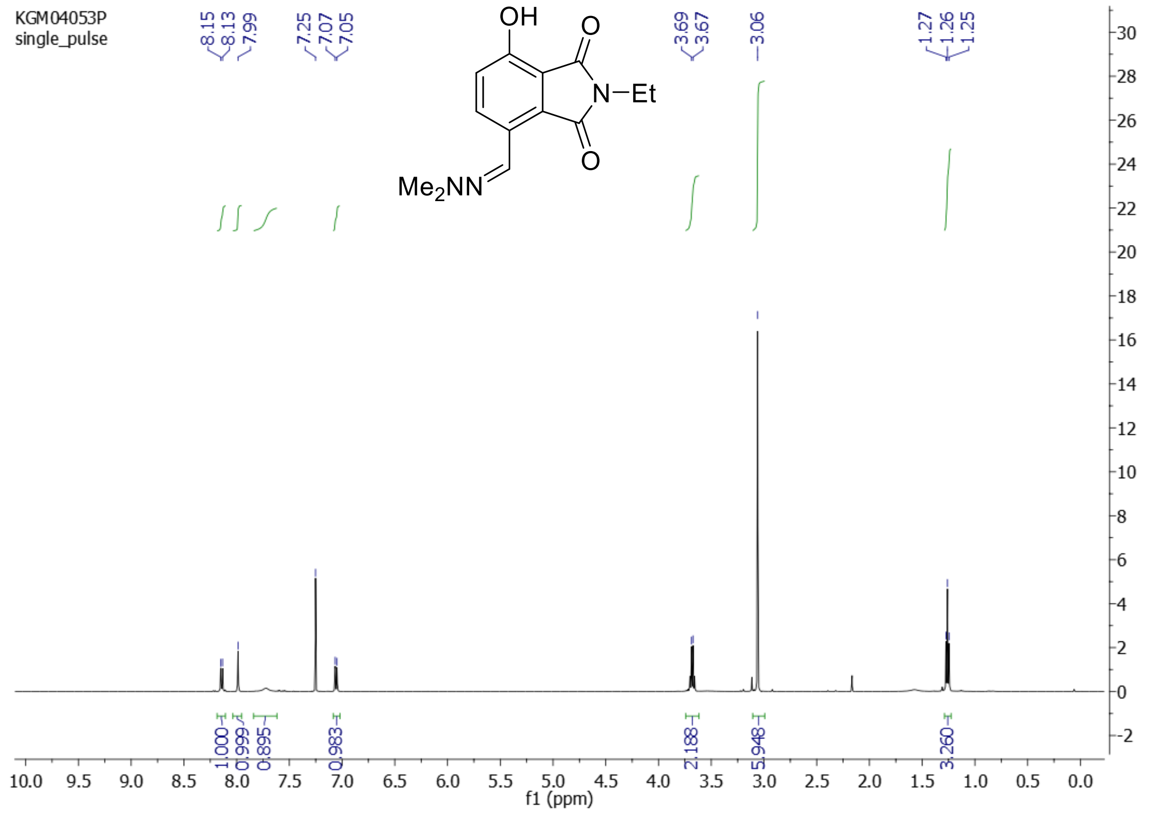

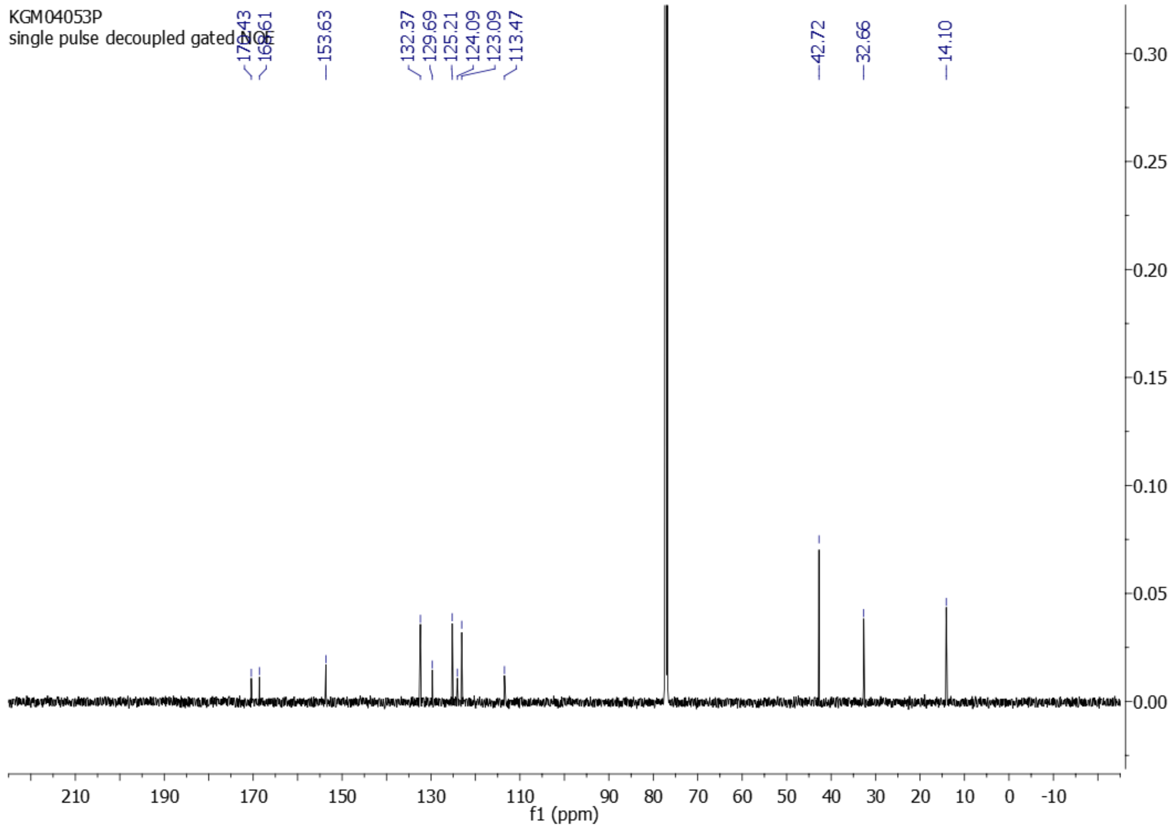

4-((2,2-Dimethylhydrazono)methyl)-7-hydroxy-2-phenylisoindoline-1,3-dione 3t

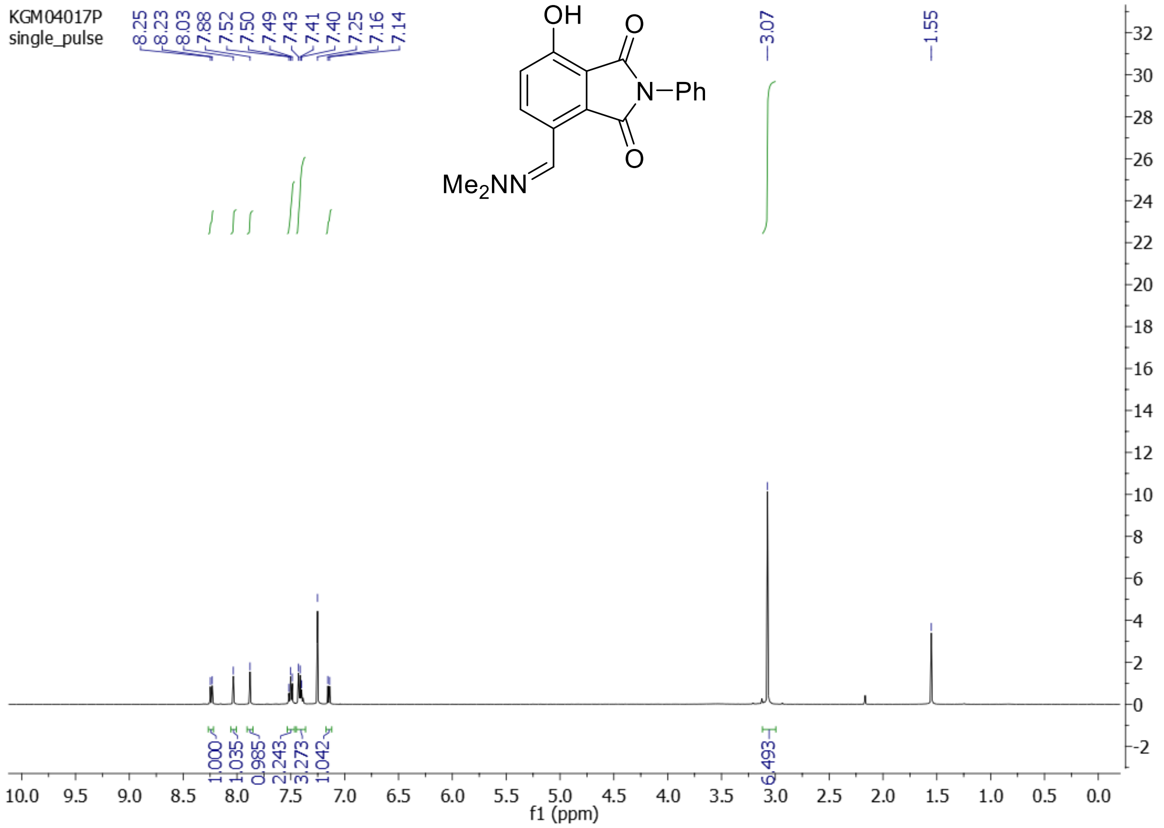

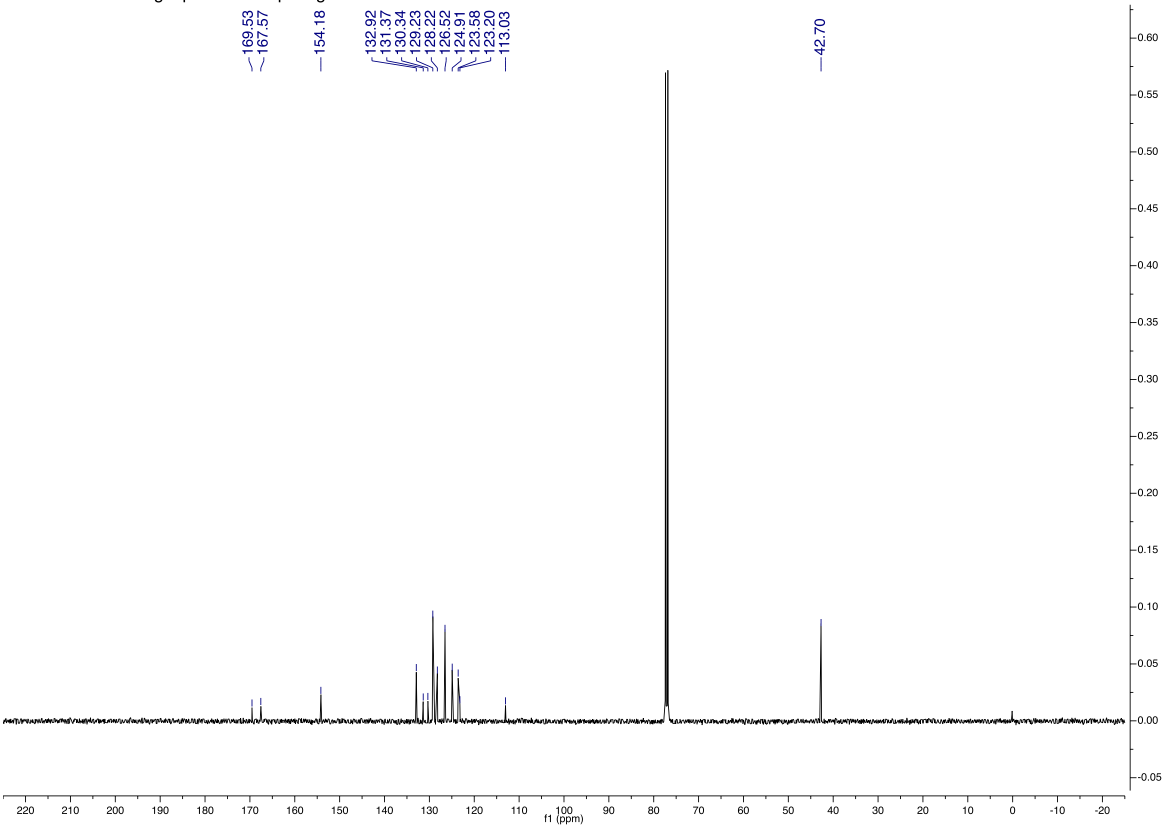

2-Furaldehyde dimethylhydrazone 1a

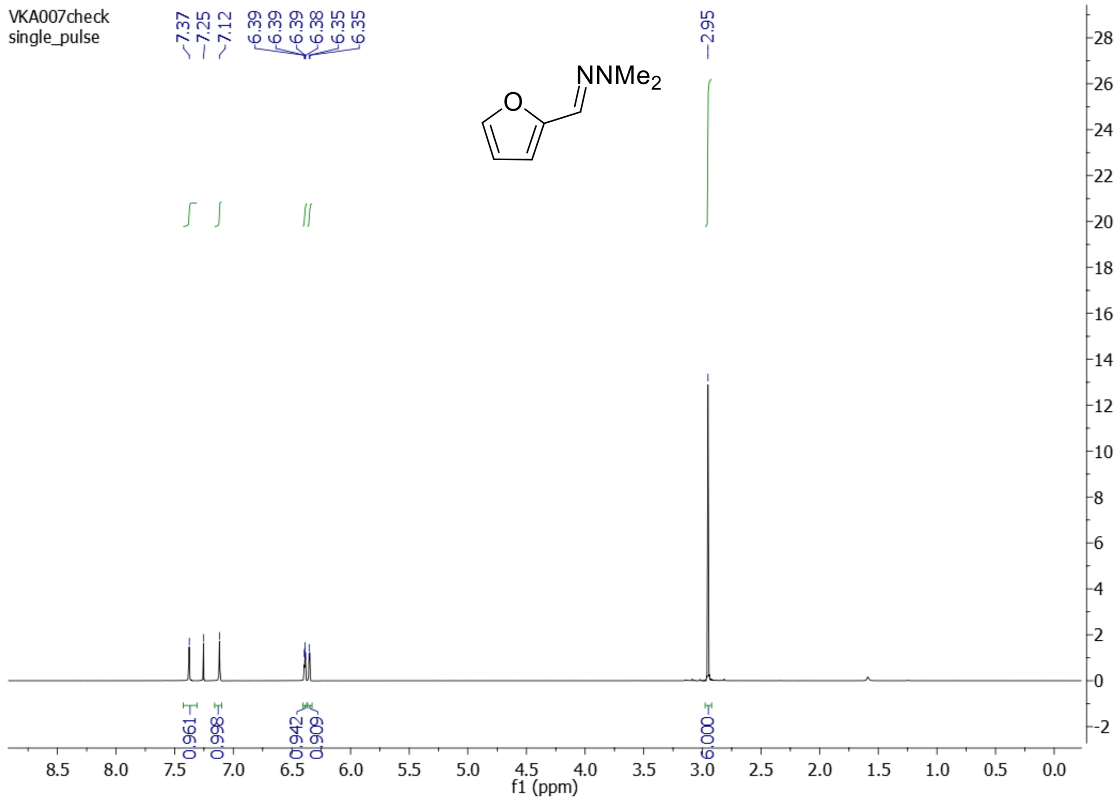

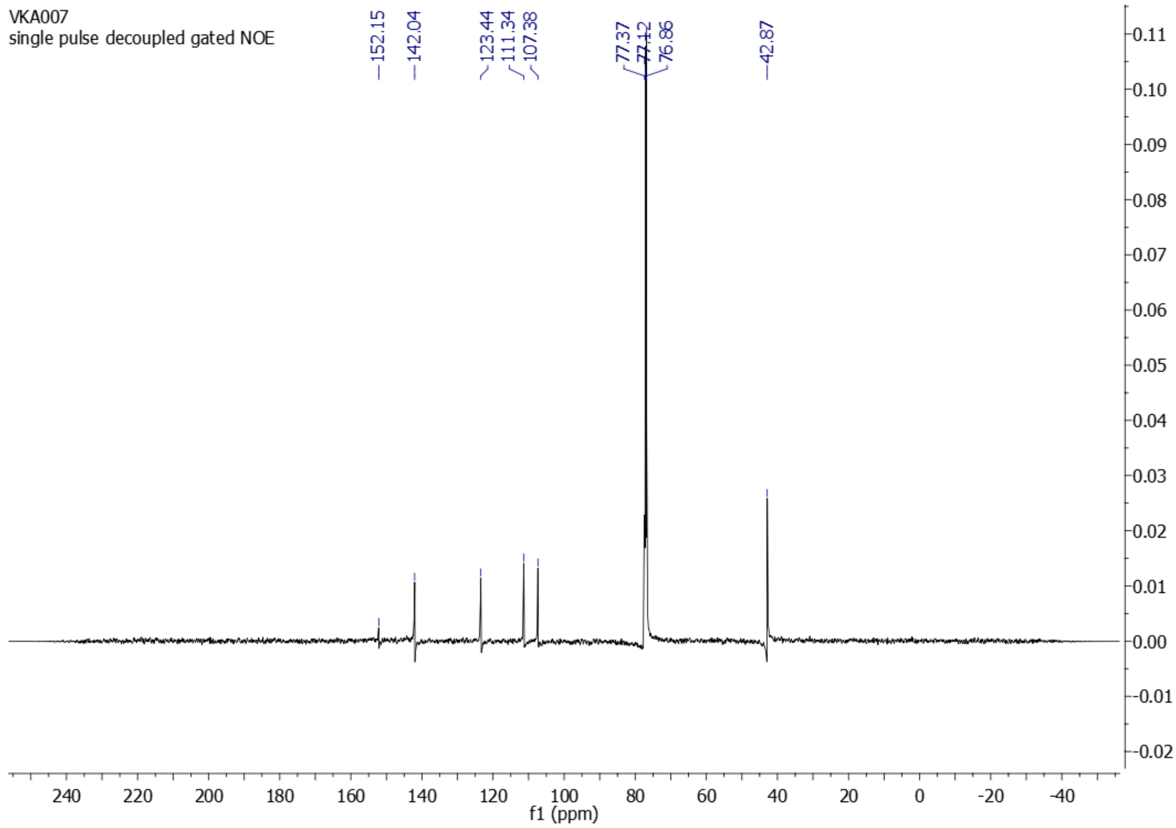

5-Methyl-2-furaldehyde dimethylhydrazone 1b

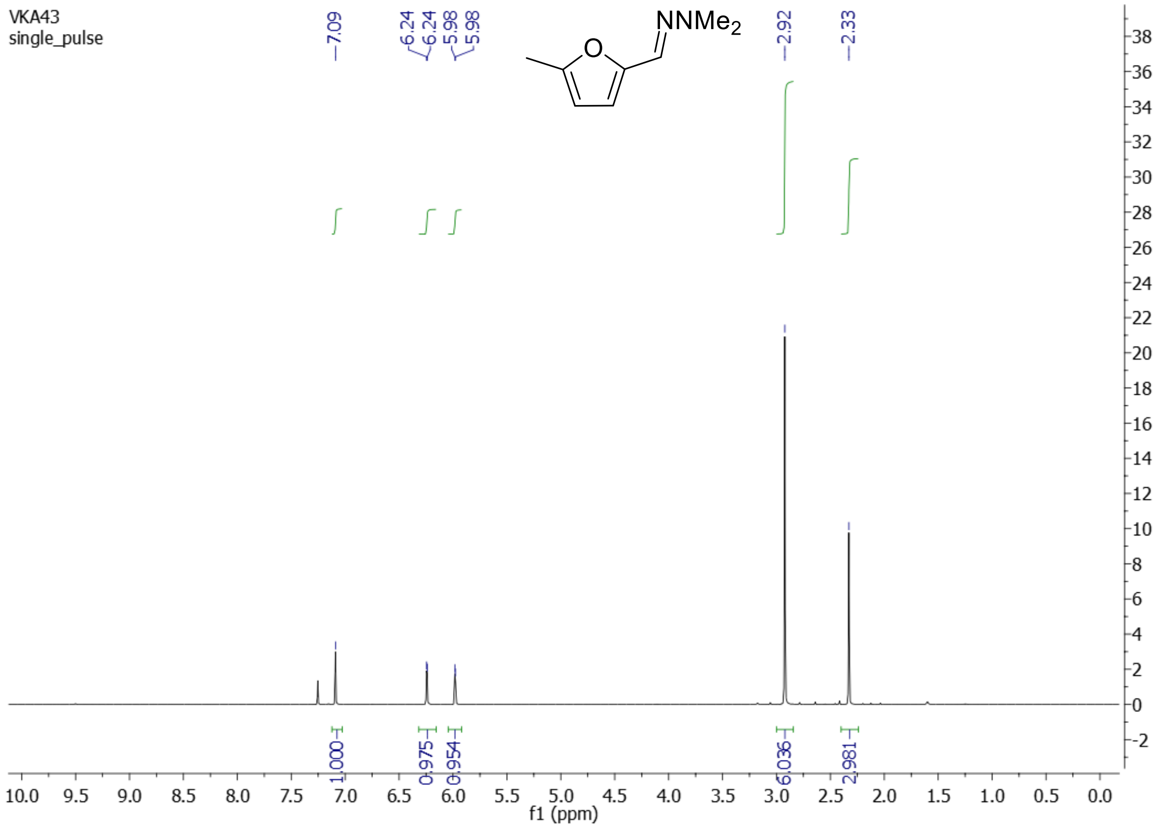

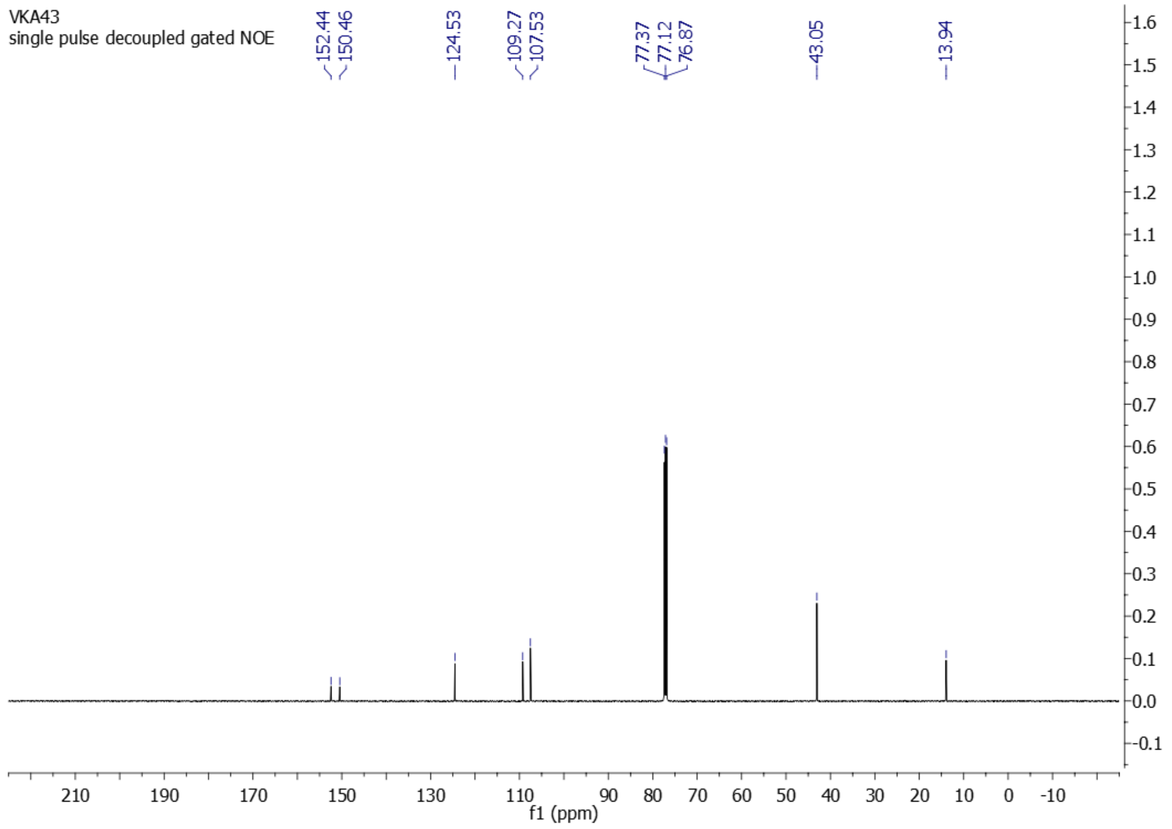

5-Bromo-2-furaldehyde dimethylhydrazone 1c

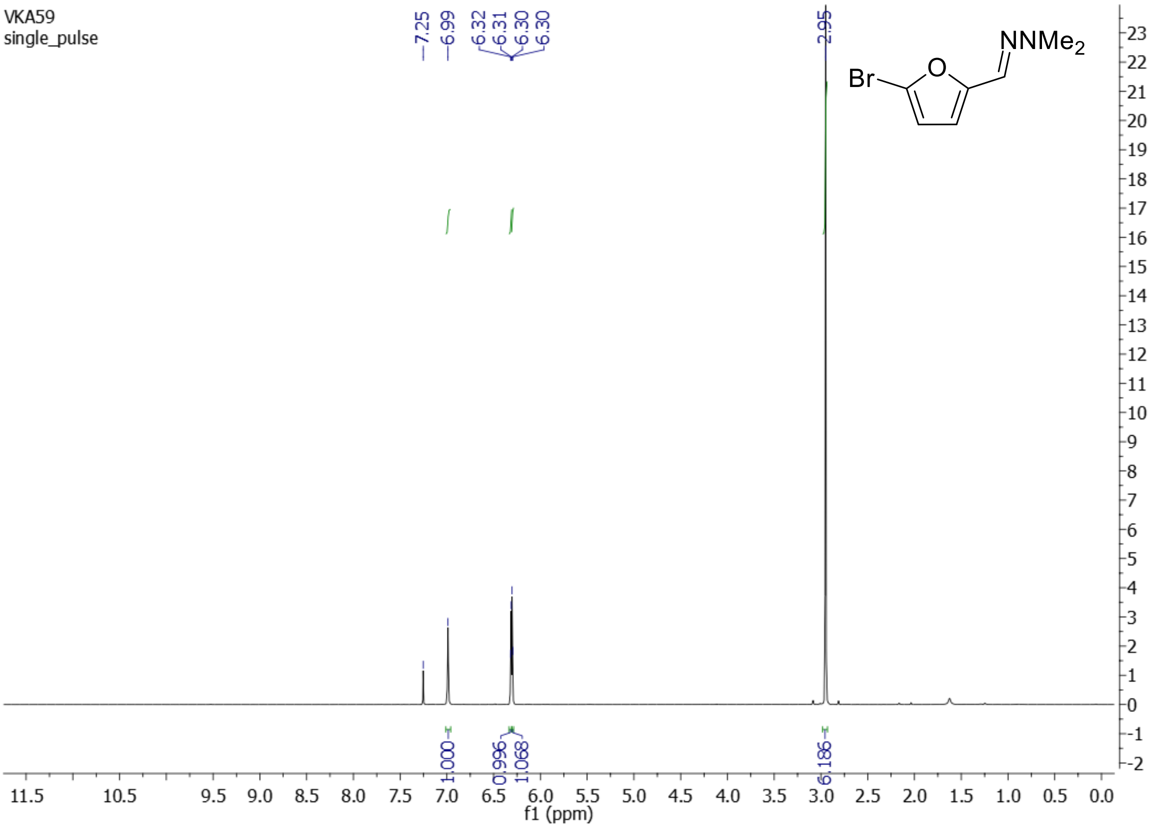

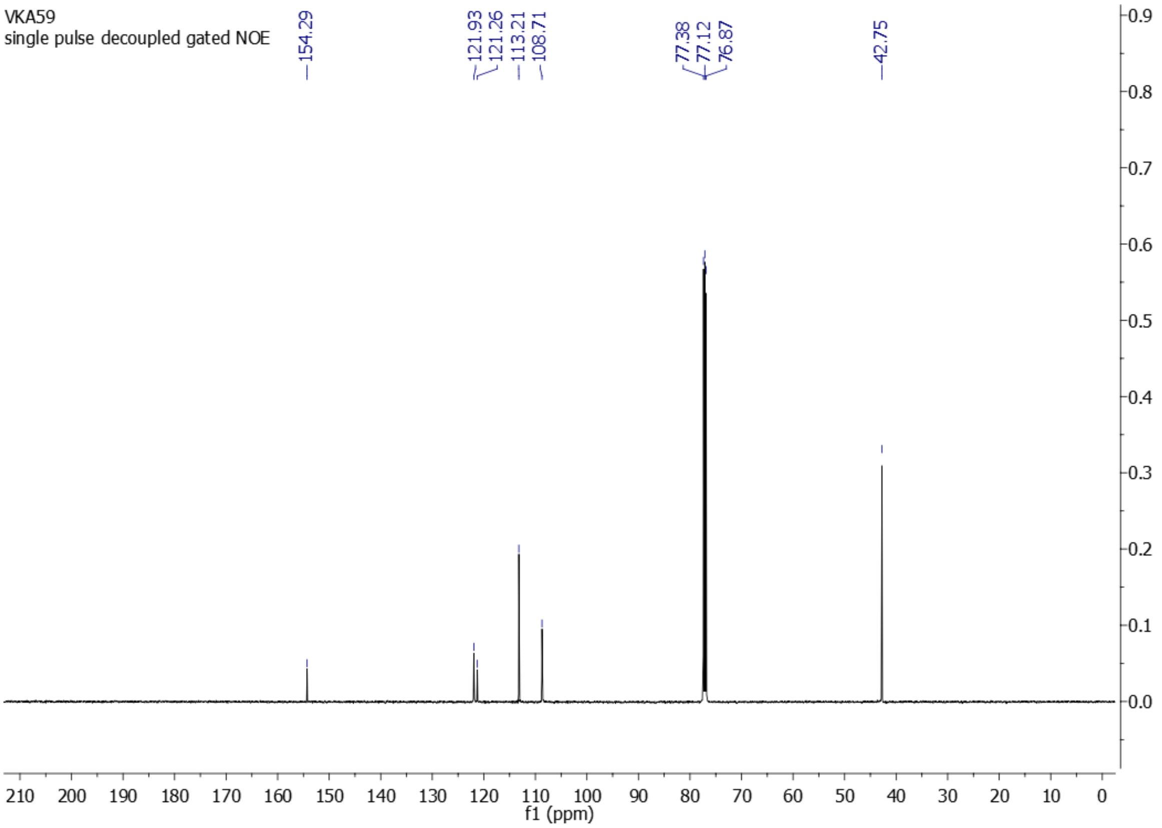

Supplement: RA-008-C8RA03895C-s001 [file RA-008-C8RA03895C-s001.pdf]
